# Estratégias de Transfusão Restritiva versus Liberal em Infarto Agudo do Miocárdio e Anemia: Metanálise e Análise Sequencial de Ensaios Clínicos

**DOI:** 10.36660/abc.20240158

**Published:** 2024-10-09

**Authors:** Ronaldo C. Fabiano, Lara Melo, Alleh Nogueira, Douglas M. Gewehr, Giuliano Generoso, Rhanderson Cardoso, Marcio S. Bittencourt

**Affiliations:** 1 University of Pittsburgh Medical Center Departmento de Clínica Médica Pittsburgh PA EUA Departmento de Clínica Médica - University of Pittsburgh Medical Center (UPMC), Pittsburgh, PA – EUA; 2 University of Connecticut Departmento de Clínica Médica Farmington CT EUA Departmento de Clínica Médica - University of Connecticut, Farmington, CT – EUA; 3 Escola Bahiana de Medicina e Saúde Pública Salvador BA Brasil Escola Bahiana de Medicina e Saúde Pública, Salvador, BA – Brasil; 4 Instituto do Coração Curitiba PR Brasil Instituto do Coração, Curitiba, PR – Brasil; 5 Universidade de São Paulo Centro de Pesquisa Clínica e Epidemiológica São Paulo SP Brasil Centro de Pesquisa Clínica e Epidemiológica - Universidade de São Paulo, São Paulo, SP – Brasil; 6 Harvard Medical School Brigham and Women's Hospital Divisão de Medicina Cardiovascular Boston MA EUA Divisão de Medicina Cardiovascular - Brigham and Women's Hospital, Harvard Medical School, Boston, MA – EUA; 7 University of Pittsburgh Medical Center Departamento de Cardiologia Pittsburgh PA EUA Departamento de Cardiologia - University of Pittsburgh Medical Center (UPMC), Pittsburgh, PA – EUA

**Keywords:** Metanálise, Infarto do Miocárdio, Anemia, Transfusão de Sangue

## Abstract

**Fundamento:**

A estratégia ótima de transfusão na anemia associada ao infarto agudo do miocárdio (IAM) ainda é desconhecida.

**Objetivos:**

Comparar a mortalidade por todas as causas entre as estratégias de transfusão liberal versus restritiva em pacientes com anemia associada a IAM, por meio de uma metanálise.

**Métodos:**

Conduzimos uma busca sistemática nos bancos de dados Pubmed, Embase, e ClinicalTrials.gov por ensaios clínicos randomizados (ECRs) comparando estratégias de transfusão liberal e restritiva na anemia associada a IAM. Uma metanálise de efeitos aleatórios e uma análise sequencial de ensaios clínicos foram conduzidas para comparar o uso de hemácias, a eficácia e desfechos de segurança. Os valores p adotados foram bicaudais, com um α de 0,05.

**Resultados:**

Em uma análise agrupada envolvendo 4217 participantes de três ECRs acompanhados por 30 dias, não foram identificadas diferenças entre as estratégias restritiva e liberal quanto a mortalidade por todas as causas (RR 1,03; IC 95% 0,67–1,57; p=0,90) e outros desfechos de eficácia (IAM recorrente, revascularização não programada, insuficiência cardíaca aguda, e lesão renal aguda), bem como desfechos de segurança incluindo reações alérgicas, infecção, e lesão pulmonar aguda. A análise sequencial dos ensaios não atingiu o limiar de futilidade. Nos pacientes alocados para a estratégia restritiva, foram observadas diferenças substanciais na transfusão utilizada entre os ECRs, correlacionadas às taxas de mortalidade, e provavelmente contribuindo para a heterogeneidade dos efeitos do tratamento entre os estudos.

**Conclusões:**

Em pacientes com anemia associada a IAM, não há uma clara superioridade entre estratégias de transfusão restritiva e liberal quanto à mortalidade por todas as causas ou outros desfechos maiores em 30 dias. No entanto, a heterogeneidade observada no uso de sangue entre os grupos submetidos à transfusão restritiva provavelmente explica a variabilidade dos achados entre os ECRs.

**Figure:**
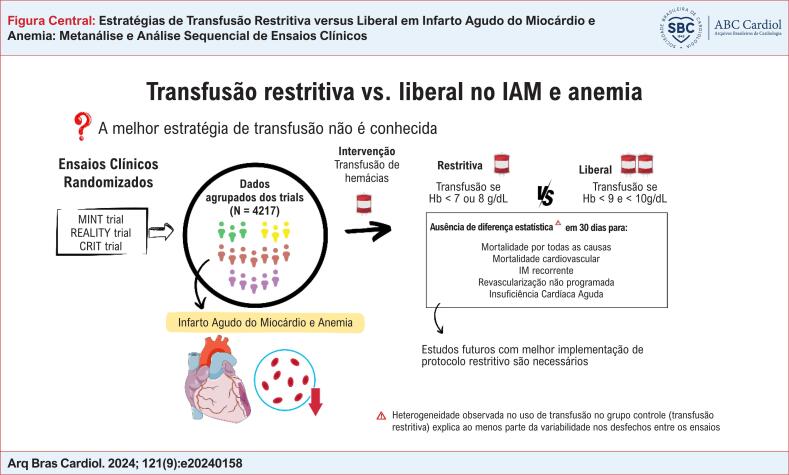

## Introdução

O limiar ótimo para transfusão de hemácias em pacientes com infarto agudo do miocárdio (IAM) e anemia continua indefinido.^1^ Evidências crescentes sugerem que não há diferença estatística na mortalidade em 30 dias nem nos desfechos clínicos importantes entre estratégias de transfusão liberal e restritiva em diversas condições clínicas.^1 – 11^

Uma estratégia de transfusão restritiva está geralmente associada a uma redução importante em transfusões de hemácias em diferentes cenários.^1^ No entanto, existe um potencial benefício clínico de uma estratégia de transfusão liberal, visando níveis mais altos de Hemoglobina (Hb) para aumentar a disponibilidade de oxigênio em pacientes com IAM e anemia. Dois Ensaios Clínicos Randomizados (ECRs) comparando limiares de transfusão restritiva e liberal em pacientes com IAM e anemia revelaram superioridade no desfecho primário composto (mortalidade intra-hospitalar, infarto recorrente, ou piora ou nova insuficiência cardíaca);^12^ e ausência de inferioridade em um evento cardiovascular adverso (morte por todas as causas, acidente vascular cerebral, infarto do miocárdio recorrente, ou revascularização de emergência)^6^ em 30 dias entre as duas estratégias.^6 , 12^

O maior e mais recente ECR até o momento, envolvendo 3504 pacientes, sugeriu uma tendência a uma mortalidade mais baixa no grupo que recebeu transfusão liberal.^13^ Dada a incerteza sobre a melhor estratégia de transfusão em pacientes com IAM e anemia e os resultados conflitantes sugeridos por esse recente ensaio,^13^ conduzimos uma revisão sistemática e metanálise comparando as estratégias de transfusão liberal *versus* restritiva nessa população ( Figura Central ).

## Métodos

Esta revisão sistemática e metanálise foi conduzida e descrita segundo o método estabelecido pela Colaboração Cochrane para revisões sistemáticas de intervenções e o *Preferred Reporting Items for Systematic Reviews and Meta-Analysis* (PRISMA) (material suplementar e métodos 1).^14 , 15^ O protocolo de metanálise foi prospectivamente registrado no registro prospectivo internacional de protocolos de revisões sistemáticas PROSPERO (CRD42023484239).^16^

### Fonte de dados e estratégia de busca

Realizamos uma busca sistemática pelos bancos de dados PubMed (MEDLINE), Embase, Cochrane, e ClincalTrials.gov desde inserção até 16 de novembro de 2023, sem restrições de idioma. Os termos buscados incluíram " *myocardial infarction* ", " *acute coronary syndrome* ", e " *blood transfusion* ". A estratégia completa de busca para cada banco de dados está apresentada no Material Suplementar e Métodos 2. Após exclusão dos artigos duplicados, dois autores (L.M. e R.F.) avaliaram títulos e resumos e avaliaram independentemente artigos completos para inclusão com base em critérios especificados previamente. Ainda, utilizamos a técnica de "bola de neve" (isto é, revisão de referências) para identificar referências relevantes de artigos identificados da busca original.^17^

#### Critérios de eligibilidade

Consideramos elegíveis para inclusão (1) ECRs; (2) que incluíram pacientes adultos (≥ 18 anos de idade) com Infarto do Miocárdio com elevação do Segmento ST (IAMST) ou Infarto do Miocárdio (IM) sem elevação do Segmento ST (IAMSSST) e anemia (Hb ≤ 10g ou Ht ≤30%); (3) que compararam estratégias de transfusão de sangue liberal vs. restritiva; e (4) apresentaram dados sobre desfechos de interesse. Os critérios de exclusão foram (1) estudos incluindo pacientes com angina estável ou instável sem estratificação de dados para aqueles com IAM; (2) IM após *bypass* da artéria coronária ou intervenção coronária percutânea; (3) pacientes recebendo tratamento paliativo; ou (4) nenhum dado sobre os desfechos de interesse.

### Extração dos dados

Dois autores (L.M. e R.F.) extraíram independentemente os dados de cada estudo usando um formulário padrão que incluía: autores, período de inclusão, ano de publicação do estudo, critérios de inclusão e exclusão, tamanho da amostra, período de seguimento, estratégias de transfusão, avaliação de IM e anemia, características basais do paciente, medicamentos usados no momento basal, dados de desfecho – número total de pacientes e número de eventos (desfechos binários), e definições de desfechos. Discordâncias foram resolvidas em um painel de discussão com um terceiro autor (A.N.).

### Desfechos

Nosso desfecho primário pré-especificado foi mortalidade por todas as causas. Os desfechos secundários de eficácia incluíram (1) mortalidade cardiovascular; (2) IM recorrente; (3) Insuficiência Cardíaca (IC) aguda; (4) acidente vascular cerebral; (5) revascularização não programada; e (6) lesão renal aguda. Nossos desfechos de segurança foram (1) lesão pulmonar aguda; (2) infecção; e (3) reação alérgica grave. Comparamos diferenças na transfusão de sangue entre os estudos e os grupos de intervenção. Definições detalhadas de cada desfecho de cada estudo incluído estão apresentadas no Material Suplementar e método 3.

### Avaliação da qualidade e risco de viés

Dois autores independentes (L.M. e R.F.) avaliaram o risco de viés nos ECRs incluídos usando o risco de viés da Cochrane (RoB2, do inglês *Risk of Bias* 2) para avaliar o risco de viés em estudos randomizados para os desfechos primários e secundários considerando os grupos com intenção de tratar.^17^ Na avaliação RoB2, a cada ensaio foi atribuído um risco alto, baixo ou incerto em cada um dos cinco domínios: viés de seleção, desempenho, detecção, atrito e de relato em relação aos desfechos primário e secundário. A avaliação GRADE ( *Grading of Recommendations, Assessment, Development, and Evaluations* ) para recomendações, avaliação, desenvolvimento e verificação da qualidade da evidência foi realizada de acordo com o manual GRADE, e o resumo dos achados foi compilado por dois autores (L.M. e R.F.) usando o programa GRADEpro;GDT).^18 , 19^ Os cinco domínios GRADE (risco de viés, inconsistência, evidência indireta, imprecisão, e viés de publicação) foram usados para categorizar o nível de certeza como alto, moderado, baixo ou muito baixo. A avaliação GRADE foi realizada para todos os desfechos relatados pelos três ensaios incluídos. Discordâncias foram resolvidas por um terceiro autor (A.N.). O teste de Egger não foi realizado dado o pequeno número de estudos incluídos (n<10), como recomendado pela Cochrane.^14^

### Análise estatística

As análises estatísticas foram conduzidas de acordo com as recomendações da Cochrane.^14^ Para acomodar heterogeneidades metodológicas e clínicas dos diferentes estudos, estimativas do efeito do tratamento foram agrupados usando um modelo de efeitos aleatórios de Mantel-Haenszel. Dado o número limitado de estudos, a estimativa da máxima verossimilhança foi usada para calcular a variância de heterogeneidade τ^2^.^14^ Desfechos binários e contínuos foram resumidos usando a razão de risco (RR) e a diferença entre as médias (DM), respectivamente, e seus respectivos intervalos de confiança de 95% (IC 95%). Os efeitos do tratamento foram bicaudais e considerados estatisticamente significativos se p<0,05. Avaliamos a heterogeneidade usando a estatística Q de Cochrane e a estatística I2 de Higgins e Thompson, com p<0,10 indicando significância estatística.^20^ Também foi realizada a análise de sensibilidade *leave-one-out* para assegurar a robustez dos nossos achados. Utilizamos o programa R, versão 4.2.2 e o pacote "meta" para os cálculos e gráficos.^21^ O código R reprodutível está disponível no Material Suplementar e métodos 4.

Para melhor avaliar potenciais erros do tipo 1 e do tipo 2, conduzimos uma Análise Sequencial de Ensaios (TSA, do inglês *trial sequential analysis* ) para mortalidade por todas as causas.^22^ Usamos um modelo de efeitos aleatórios com IC 95%, um eixo de informação com tamanho amostra, erro tipo 1 com limiar bicaudal de 5%, e poder de 80%. O ajuste dos limiares para o escore Z foi baseado na função de consumo de O'Brien–Fleming. A TSA foi realizada usando o programa TSA versão 0.9.5.10 beta (Copenhagen Trial Unit, Centre for Clinical Intervention Research, Rigshospitalet, Copenhagen, Dinamarca).^23^

## Resultados

### Seleção dos estudos e características basais

**Figura 1:**
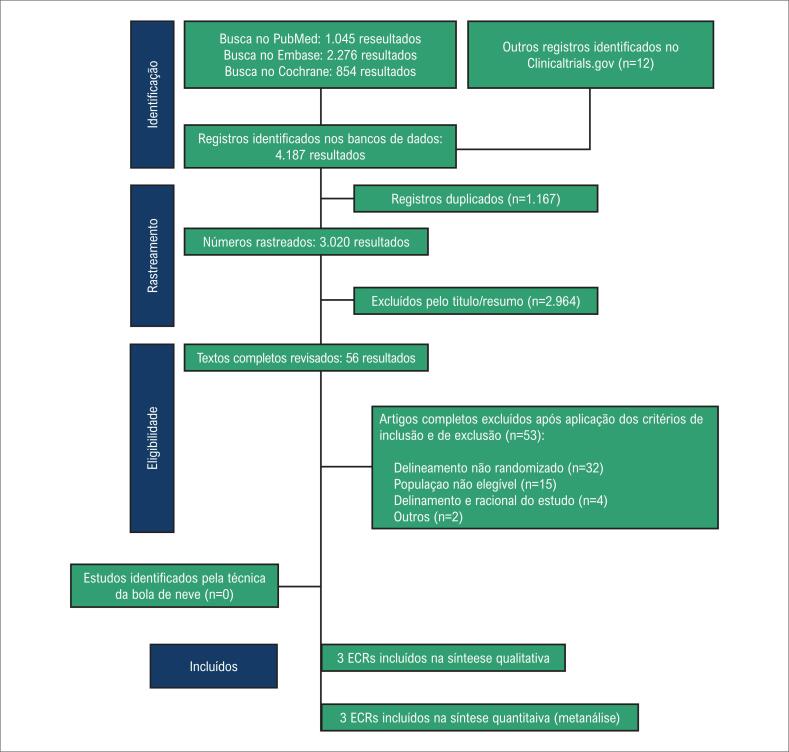
Rastreamento e seleção dos estudos.

A busca sistemática gerou 4187 artigos e resumos ( Figura 1 ). Após remoção das duplicatas e estudos que preencheram os critérios de exclusão com base na revisão de títulos e resumos, 56 artigos foram identificados e revisados na íntegra para possível inclusão. Finalmente, quatro artigos preencheram os critérios de inclusão e foram analisados, mas um estudo foi removido dada a ausência de dados estratificados para os pacientes com IAM, uma vez que este estudo também incluiu pacientes sem IAM.^24^ As razões para as exclusões na revisão dos artigos completos estão detalhadas na eTabela 1 do material suplementar. Incluímos 4217 pacientes, com 2115 (50,2%) alocados para receberem transfusão sanguínea restritiva e 2100 (49,8%) alocados para a estratégia de transfusão liberal. A idade média dos pacientes foi 72,8 anos (72,1-77,0 anos), e 45% (42,2-48,0%) eram do sexo feminino ( Tabela 1 ). Em todos os três estudos, os desfechos de interesse foram avaliados em 30 dias. Definições das estratégias de transfusão liberal e restritiva em cada estudo estão detalhadas na eTabela 2 do material suplementar. As características clínicas iniciais dos pacientes incluídos estão resumidas na eTabela 3 do material suplementar.

**Tabela 1:** Características basais dos estudos e dos pacientes incluídos

Características do estudo		MINT, 2023 (N = 3504)		REALITY, 2021 (N = 668)		CRIT, 2011 (N = 45)	
Principais critérios de inclusão		Idade ≥ 18 anos IAM Hb <10 g/dL		Idade ≥ 18 anos IAM Hb 7-10 g/dL		IAM Ht ≤ 30% em 72 horas do início dos sintomas	
		Restritiva (N =1749)	Liberal (N =1755)	Restritiva (N =342)	Liberal (N =324)	Conservadora (N =24)	Liberal (N =21)
Estratégia de transfusão, (%)		Hb 7 ou 8 g/dL, (50)	Hb <10 g/dL, (50)	Hb <8 g/dL, (51)	Hb <10 g/dL, (49)	Ht <24%, (53)	Ht <30%, (47)
Idade em anos, média ± DP		72,2 ±11,5	72,1 ±11,6	78 (69-85)	76 (69-84)	70,3 ±14,3	76,4 ±13,5
Sexo feminino, No. (%)		774 (44,3)	819 (46,7)	141 (41,2)	140 (43,2)	11 (46)	11 (52)
Hipertensão, No. (%)		1478 (84,5)	1498 (85,4)	272 (79,5)	256 (79,0)	18 (75)	19 (91)
Dislipidemia, No. (%)		1123 (64,2)	1147 (65,4)	189 (55,3)	201 (62,0)	15 (63)	16 (76)
Diabetes, No. (%)		948 (54,2)	948 (54,0)	176 (51,5)	158 (48,8)	13 (54)	17 (81)
Tabagismo atual, No. (%)		273 (16,6)	275 (16,6)	51 (16,1)	41 (14,0)	8 (33)	2 (10)
**Histórico de eventos (antes do índice) N. (%)**							
	IM	589 (33,7)	549 (33,1)	121 (35,4)	119 (36,7)	NA	NA
	ICP	623 (35,6)	577 (32,9)	114 (33,3)	111 (34,3)	6 (25)	5 (24)
	CABG	372 (21,3)	390 (22,2)	44 (12,9)	42 (13,0)	4 (17)	6 (29)
	Insuficiência Cardíaca Aguda	527 (30,1)	539 (30,7)	44 (12,9)	38 (11,7)	NA	NA
	Anemia crônica	735 (42,0)	758 (43,2)	61 (17,8)	62 (19,1)	NA	NA
	Câncer	397 (22,7)	372 (21,2)	67 (19,5)	62 (19,1)	NA	NA
	DRT	797 (45,6)	810 (46,2)	25 (7,3)	30 (9,3)	NA	NA
**IAM índice, N. (%)**							
	IAMSSST	1430 (81,8)	1418 (80,8)	234 (68,4)	231 (71,3)	13 (54)	14 (67)
	STEMI	319 (18,2)	337 (19,2)	108 (31,6)	93 (28,7)	11 (46)	7 (33)
**Achados antes da randomização**							
FEVE %, média ± DP		47,3 ±13,4	47,5 ±13,7	ND	ND	39 ±15	47 ±13
Creatinina, mediana (Q1, Q3) ou média ± DP		1,4 (0,9, 2,6)	1,4 (0,9, 2,5)	1,3 (0,9, 2,0)	1,2 (0,9, 2,2)	2,4 ± 2,3	2,9 ± 2,3
Hb * , média ± DP		8,6 ±0,8	8,6 ±0,8	9,0 ±0,8	9,1 ±0,8	NA	NA
Sangramento ativo, N. (%)		246 (14,1)	213 (12,1)	36 (10,5)	49 (15,1)	NA	NA
Hemácias (bolsas), média ± DP		0,7 ±1,6	2,5 ±2,3	2,9 ±3,7	2,8 ±2,7	1,6 ± 2,0	2,5 ±1,3

* Hb no ensaio CRIT foi estimada como Ht/3.^39^ Todos os estudos adotaram um p< 0,05 como significância estatística; IAM: infarto agudo do miocárdio; CABG: bypass da artéria coronária; DRT: doença renal terminal; Hb: hemoglobina; ND: não disponível; FEVE: fração de ejeção do ventrículo esquerdo; IM: infarto do miocárdio; IAMSSST: infarto agudo do miocárdio sem elevação do Segmento ST; ICP: intervenção coronária percutânea; Q1 e Q3, primeiro e terceiro quartis; ECR: ensaio clínico randomizado; DP: desvio padrão; IAMSST: infarto agudo do miocárdio com elevação do segmento ST.

### Desfechos de eficácia

**Figura 2:**
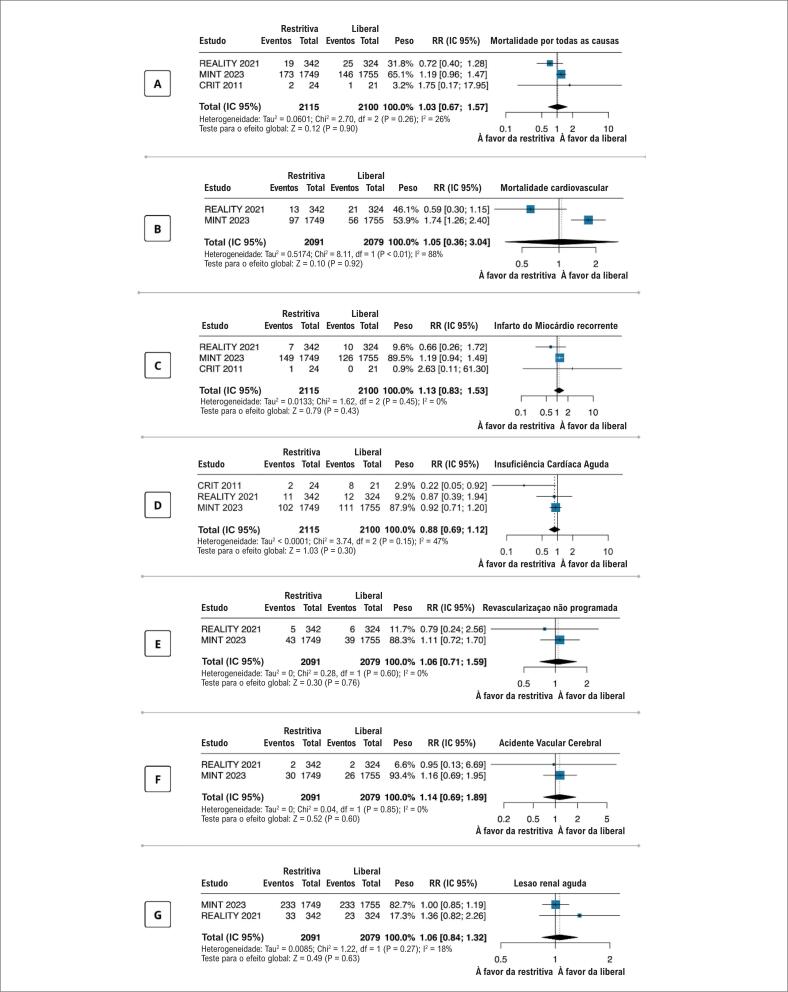
Desfechos da estratégia de transfusão restritiva versus transfusão liberal em pacientes com infarto agudo do miocárdio e anemia em 30 dias; (A) mortalidade por todas as causas; (B) infarto agudo do miocárdio recorrente; (C) infarto agudo do miocárdio recorrente; (D) insuficiência cardíaca aguda; (E) revascularizações não programadas; (F) acidente vascular cerebral; (G) lesão renal aguda; CRIT: Conservative Versus Liberal Red Cell Transfusion in Acute Myocardial Infarction (the CRIT Randomized Pilot Study);12 MINT: Restrictive or Liberal Transfusion Strategy in Myocardial Infarction and Anemia;13 REALITY: Effect of a Restrictive vs Liberal Blood Transfusion Strategy on Major Cardiovascular Events Among Patients With Acute Myocardial Infarction and Anemia;6 todos os estudos empregaram um valor de p< 0,05 como significância estatística.

Em pacientes com anemia associada a IAM, não houve diferenças estatisticamente significativas entre as estratégias restritiva e liberal para mortalidade por todas as causas ( Figura 2A ), mortalidade cardiovascular ( Figura 2B ), IM recorrente ( Figura 2C ), IC aguda ( Figura 2D ), revascularização não programada ( Figura 2E ), acidente vascular cerebral ( Figura 2F ), e lesão renal aguda ( Figura 2G ).

Os resultados foram robustos e consistentes com os resultados primários quando a análise *leave-one-out* foi conduzida para mortalidade por todas as causas, IM recorrente, e IC aguda (Material suplementar eFigura 1 ).

**Figura 3:**
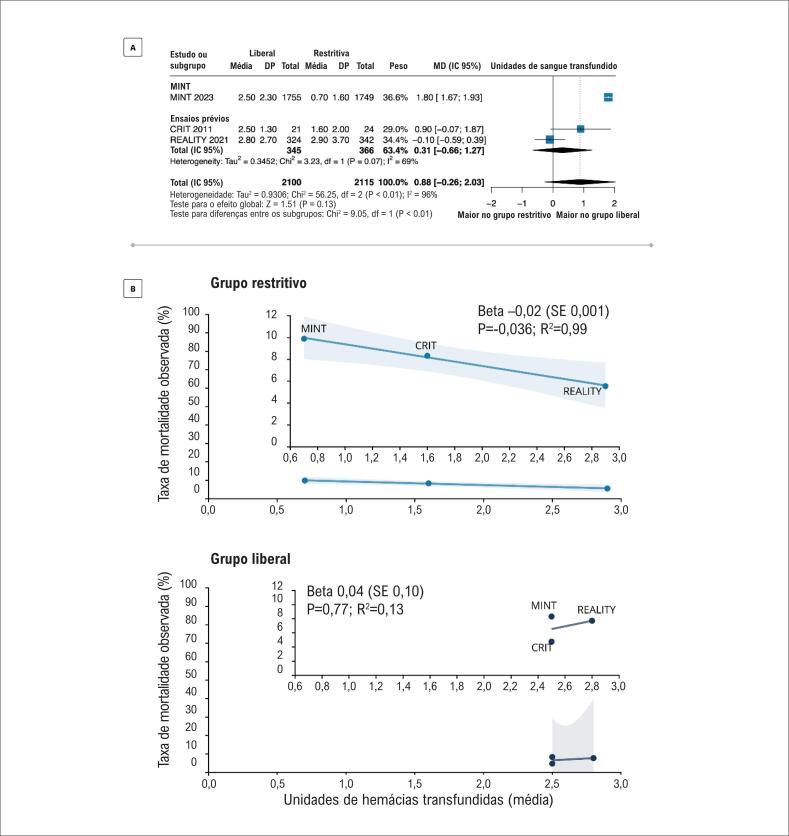
Diferença entre as médias de unidades de hemácias transfundidas (g/dL) (A) e tendência de mortalidade (B) na estratégia de transfusão restritiva versus estratégia de transfusão estratégia de transfusão liberal em pacientes com infarto agudo do miocárdio e anemia em 30 dias; CRIT: Conservative Versus Liberal Red Cell Transfusion in Acute Myocardial Infarction (the CRIT Randomized Pilot Study);^12^ MINT: Restrictive or Liberal Transfusion Strategy in Myocardial Infarction and Anemia;^13^ REALITY: Effect of a Restrictive vs Liberal Blood Transfusion Strategy on Major Cardiovascular Events Among Patients With Acute Myocardial Infarction and Anemia; todos os estudos empregaram um valor de p< 0,05 como significância estatística.

Em uma avaliação da heterogeneidade entre os estudos, nós avaliamos a transfusão total usada em cada estudo. Enquanto o grupo da estratégia liberal mostrou um uso similar de transfusão em todos os ensaios, observou-se uma alta heterogeneidade de tratamento no grupo da estratégia restritiva. O número médio de unidades de hemácias nos ensaios MINT, CRIT e REALITY foram 0,7 (±1,6), 1,6 (±2,0), e 2,9 (±3,7) respectivamente, no grupo restritivo ( Tabela 1 , Figura 3A ).^6 , 12 , 13^ No último estudo, vale destacar que o número total de unidades de bolsas de hemácias administradas no grupo restritivo foi ainda maior que no grupo da estratégia liberal (341 vs. 324) ( Figura 3A ).^6^ Esse resultado está significantemente associado com os desfechos dos estudos uma vez que observou-se uma mortalidade mais alta nos estudos que prescreveram um número menor de bolsas de hemácias no grupo restritivo ( Figura 2 B ). Esse achado provavelmente explica a heterogeneidade entre os estudos.

### Desfechos de segurança

**Figura 4:**
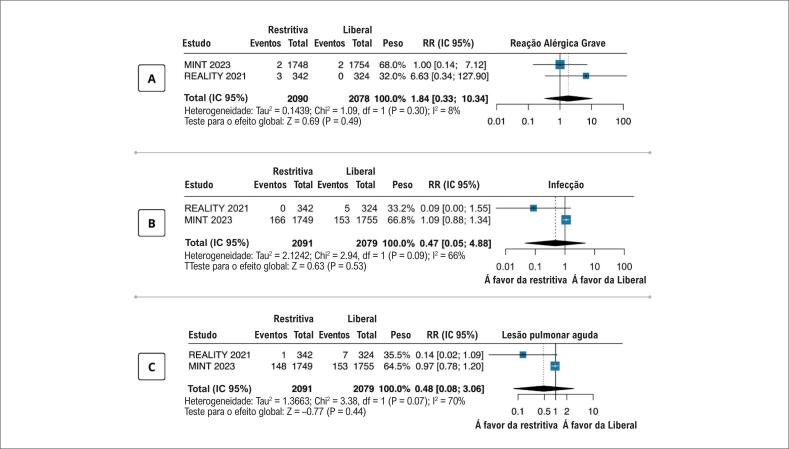
Desfechos de segurança da estratégia de transfusão restritiva versus estratégia de transfusão liberal em pacientes com infarto agudo do miocárdio e anemia em 30 dias; reações alérgicas (A), infecções (B), e lesão pulmonar aguda ou insuficiência respiratória (C); CRIT: Conservative Versus Liberal Red Cell Transfusion in Acute Myocardial Infarction (the CRIT Randomized Pilot Study);^12^ MINT: Restrictive or Liberal Transfusion Strategy in Myocardial Infarction and Anemia;^13^ REALITY, Effect of a Restrictive vs Liberal Blood Transfusion Strategy on Major Cardiovascular Events Among Patients With Acute Myocardial Infarction and Anemia; todos os estudos empregaram um valor de p< 0,05 como significância estatística.

Não foram observadas diferenças entre os grupos quanto às reações alérgicas graves, infecção, e lesão pulmonar aguda ou insuficiência respiratória ( Figura 4 ).

### Avaliação da qualidade e risco de viés

O risco de viés para cada um dos cinco domínios analisados (seleção, desempenho, detecção, atrito e vieses de relato) foi baixo e concordante entre os dois autores (L.M. e R.F.). O risco foi avaliado pela ferramenta RoB2 para os desfechos primários e secundários considerando os grupos com intenção de tratar para cada um dos três ensaios incluídos, resultando em um risco total de viés para cada estudo (Material Suplementar eTabela 4 ).

O nível final de certeza de evidência para o efeito estimado agrupado (restritivo vs. liberal) foi moderado para mortalidade por todas as causas, IM recorrente, e IC aguda – desfechos relatados em todos os ECRs incluídos. Para cada um desses desfechos, a imprecisão provocou a diminuição na certeza estatística dado que o potencial benefício e o potencial malefício de transfusão ficou dentro do intervalo de confiança (Material Suplementar eTabela 5 ).

### Análise sequencial de ensaios

**Figura 5:**
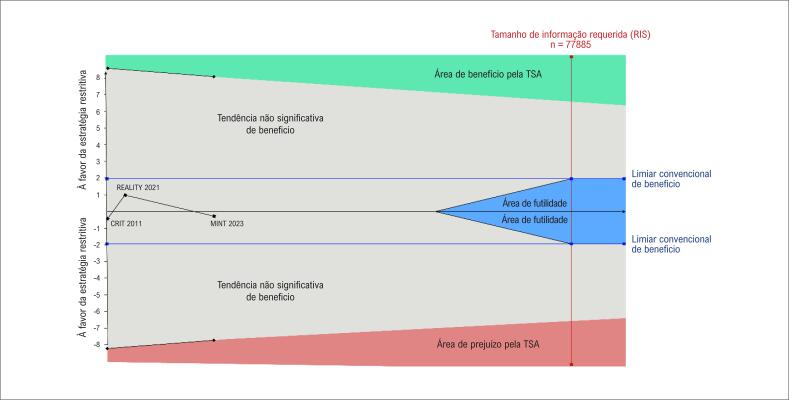
Análise Sequencial de Ensaios da estratégia de transfusão restritiva versus estratégia de transfusão liberal da mortalidade por todas as causas em 30 dias em pacientes com infarto agudo do miocárdio e anemia; CRIT: Conservative Versus Liberal Red Cell Transfusion in Acute Myocardial Infarction (the CRIT Randomized Pilot Study); MINT: Restrictive or Liberal Transfusion Strategy in Myocardial Infarction and Anemia; REALITY: Effect of a Restrictive vs Liberal Blood Transfusion Strategy on Major Cardiovascular Events Among Patients With Acute Myocardial Infarction and Anemia.

Na TSA da mortalidade por todas as causas, a curva-Z cumulativa não ultrapassou os limiares de monitoramento, incluindo futilidade. Ainda, o tamanho da amostra total não atingiu o tamanho da informação necessária ( Figura 5 ). Em geral, esses resultados indicam ausência de diferença estatisticamente significativa na análise agrupada e, até o momento, não são suficientes para excluir definitivamente a possibilidade de um efeito de uma estratégia de transfusão liberal vs. transfusão restritiva para pacientes com IAM e anemia.

## Discussão

No presente estudo, não encontramos diferenças estatisticamente significativas em desfechos de 30 dias entre uma estratégia de transfusão liberal *versus* restritiva para mortalidade por todas as causas, mortalidade cardiovascular, IM recorrente, revascularização não programada, IC, acidente vascular cerebral e lesão renal aguda em pacientes com IM agudo e anemia. De maneira similar, não houve diferenças significativas nos desfechos de segurança. Esses resultados continuaram robustos nas análises de sensibilidade *leave-one-out* para todos os desfechos. Contudo, observou-se uma heterogeneidade notável no resultado entre os estudos, o que parece ser ao menos em parte explicada pelas diferenças nas quantidades de bolsas de hemácias usadas nos grupos submetidos à estratégia restritiva nos diferentes ensaios.

A abordagem restritiva reduz o uso de um recurso crucial e limitado, bem como os riscos potenciais de efeitos colaterais associados. Porém, o suposto benefício de se manter os níveis de Hb mais elevados para aumentar a disponibilidade de oxigênio para a área de isquemia do miocárdio é plausível e justifica maiores investigações, embora alguns estudos sugiram que a oferta de oxigênio pode não ser aumentada por transfusões.^25^ Evidências sugerem que um estratégia de transfusão restritiva é segura em uma gama de cenários clínicos diferentes,^1^ e nossa análise corrobora isso ao documentar ausência de diferenças em qualquer dos desfechos de segurança entre os dois grupos.

Enquanto nenhuma diferença notável nos desfechos de segurança sugeriria que uma das duas estratégias seria a mais aceitável para uso de rotina, a logística de produtos hemoderivados é mais complexa que a maioria das terapias usadas rotineiramente. Os recursos são escassos, e qualquer potencial redução no seu uso pode ter um impacto significativo a partir de uma perspectiva social, uma vez que esses recursos podem ser direcionados a outros pacientes que os necessitam.^26 – 28^ Essa realocação de recursos escassos também pode levar a uma economia de gastos e a melhorias logísticas na implementação de seu uso.^26^ Assim, a menos que exista um benefício comprovado de estratégias de transfusão mais liberais, uma estratégia restritiva provavelmente ofereceria mais benefício à sociedade como um todo, já que não foi observado um impacto clínico importante nos pacientes individualmente.

No entanto, dado o profundo impacto desses resultados na prática clínica em unidades de terapia intensiva em todo o mundo, é necessária uma análise mais detalhada para uma implementação mais adequada da evidência atual. Nesse contexto, nossos resultados provêm novos achados esclarecedores na avaliação da heterogeneidade entre os estudos incluídos na presente metanálise. Enquanto o tratamento na estratégia foi surpreendentemente consistente entre os ensaios, houve uma grande variabilidade no uso de transfusão no grupo definido como "grupo de transfusão restritiva". Essa diferença não foi trivial, uma vez que variou de uma média de uma unidade de bolsa de hemácias por paciente a quase três unidades. Parte da heterogeneidade pode ser explicada pelas metas de Hb a ser atingidas após a transfusão no grupo restritivo entre os ensaios. No ensaio MINT,^13^ não foi necessária transfusão quando a Hb era inferior a 8g/dL e, consequentemente, esse foi o ensaio com a menor média de unidades de sangue transfundidas. Por outro lado, o ensaio REALITY^6^ tinha o alvo mais alto após a transfusão no grupo restritivo (98-10g/dL), o que se aproxima ao alvo pós-transfusão no grupo liberal (≥ 10 g/dL) nos ensaios MINT e CRIT.^12 , 13^

Outro ponto a ser considerado é a duração do seguimento. No ensaio REALITY,^6^ apesar do benefício observado na estratégia restritiva em curto prazo, os achados positivos não foram mantidos em um ano de seguimento.^29^ Esse resultado reforça a necessidade de um período de seguimento sequencial bem definido, estratificado por populações específicas. Até lá, o limiar para a transfusão deve ser individualizado, levando em consideração o contexto clínico do paciente.

A diferença na transfusão entre os dois grupos em cada ensaio é um parâmetro chave a ser explorado, uma vez que a eficácia de uma terapia só pode ser comprovada se o seu uso for consistentemente e significativamente diferente entre os dois grupos de estudo. Se o grupo controle (restritivo) receber quase a mesma quantidade de transfusões que o grupo tratamento (liberal), não serão esperadas diferenças nos desfechos. Em nossa análise, conseguimos demonstrar uma correlação direta entre a transfusão no braço controle e os desfechos observados (redução na taxa de mortalidade com um aumento na média de unidades de sangue transfundido no grupo restritivo). Embora a análise seja limitada pelo pequeno número de estudos (três), o pequeno tamanho amostral leva principalmente a um poder consideravelmente menor na análise; porém, isso não teria um impacto substancial sobre a taxa de resultados falso positivos.

Existem outras explicações potenciais para os achados que não fomos capazes de explorar, tais como a heterogeneidade entre os pacientes com IAM e anemia, além da impossibilidade de se considerar a Hb como um substituto ótimo para a disponibilidade de oxigênio.^30 – 34^ Ainda, o IAM engloba tanto pacientes com IAMSST como pacientes com IAMSSST, os quais geralmente exibem diferentes cargas isquêmicas, gravidade clínica, e prognóstico em 30 dias, contribuindo para uma heterogeneidade significativa dentro do trupo.^30^ Similarmente, os indivíduos com anemia aguda e crônica podem passar por diferentes adaptações fisiológicas à isquemia e à transfusão sanguínea, incluindo variações na curva de dissociação da oxi-hemoglobina.^31 , 34^ Nesse mesmo contexto, alterações nas hemácias, coletivamente chamadas como "lesões de armazenamento", podem impactar de maneira diferente o transporte de oxigênio pelas hemácias e a oferta de oxigênio no tecido, incluindo implicações fisiológicas potencialmente distintas de diferentes doadores das hemácias.^35 , 36^ Ainda, é plausível que pacientes com doença renal crônica concomitante possam ter uma resposta diferente à isquemia e à transfusão.^34 , 37^ Finalmente, dada a natureza heterogênea da anemia associada ao IAM e possíveis efeitos desequilibrados complexos na própria intervenção (transfusão sanguínea), essas duas estratégias de transfusão podem ter efeitos diferentes e opostos nos subgrupos distintos, o que pode não ser completamente desejável dado o poder limitado das análises dos subgrupos.^38^ Apesar de todas essas explicações alternativas para os achados serem plausíveis e potencialmente importantes, nenhuma delas contradiz os achados do presente estudo.

Quando esses resultados de heterogeneidade são contextualizados com nossa TSA, torna-se claro que as evidências existentes são insuficientes para corroborar o uso de uma das duas estratégias. Contudo, nossos resultados dão um direcionamento sobre como estudos futuros devam ser conduzidos para fornecer informações significativas para implementação dos resultados. Esses estudos devem não só focar na implementação adequada da estratégia de tratamento no braço liberal, mas também implementar, de modo controlado, a estratégia de transfusão no braço restritivo, para se obter uma diferença real de tratamento entre os grupos. Isso gera dúvidas sobre caso houvesse uma diferença significativa entre as duas estratégias, seu efeito provavelmente seria pequeno. No entanto, essa análise é limitada pela análise cumulativa dos dados de todos os ensaios incluídos. Se uma diferença entre tratamentos fosse assumida, como aquela relatada no ensaio MINT (um aumento em três vezes na quantidade de sangue utilizado), as diferenças calculadas nos desfechos relevariam a necessidade de um tamanho amostral muito menor.

Este estudo tem limitações. Primeiro, todos os três ECRs eram abertos dada a natureza da intervenção (transfusão de sangue). Segundo, a ausência de dados individuais dos pacientes não permitiu análises subgrupos específicos. E terceiro, os três estudos empregaram metas de transfusão ligeiramente diferentes.

## Conclusão

Nesta metanálise de ECRs de pacientes com IAM e anemia, não houve diferença estatisticamente diferente entre a estratégia de transfusão restritiva e a estratégia de transfusão em 30 dias quanto à mortalidade por todas as causas, mortalidade cardiovascular, IM recorrente, revascularização não programada, insuficiência cardíaca aguda, lesão renal aguda, reação alérgica grave, infecção, lesão pulmonar aguda, e insuficiência respiratória. Entretanto, a heterogeneidade observada no uso de transfusão nos grupos controles (transfusão restritiva) provavelmente explica ao menos parte da variabilidade nos desfechos entre os ensaios. Os resultados direcionam a implementação de ensaios futuros para explorar essa questão.

## *Material suplementar

Para informação adicional, por favor, clique aqui .
