# Supplementary material for: Estratégias de Transfusão Restritiva versus Liberal em Infarto Agudo do Miocárdio e Anemia: Metanálise e Análise Sequencial de Ensaios Clínicos
Source: Arq Bras Cardiol. 2024 Oct 9;121(9):e20240158. [Article in Portuguese] doi: 10.36660/abc.20240158 (PMC12092027; doi:10.36660/abc.20240158)
Supplement: Supplementary file 1 [file 0066-782X-abc-121-09-e20240158-Suppl01.pdf]

SUPPLEMENT MATERIAL

Restrictive versus Liberal Transfusion Strategies in Acute Myocardial Infarction and Anemia: A Meta-Analysis and Trial Sequential Analysis of Randomized Controlled Trials

Table of Contents

Supplement and Methods 1. PRISMA Main Checklist[1] 3
Supplement and Methods 2. Details of the Search Strategy According to the Database 6
Supplement and Methods 3. Definition of endpoints 7
Supplement and Methods 4. Reproducible code on R software for calculations and graphics[5] 9
Supplement and Table 1. Detailed reason for exclusion in full-text review 17
Supplement and Table 2. Definition of restrictive and liberal transfusion strategies 20
Supplement and Table 3. Clinical baseline characteristics of the included trials 21
Supplement and Figure 1. Leave-one-out analysis for all-cause mortality (A), heart failure (B), and recurrent myocardial infarction (C)[58] 25
Supplement and Table 4. Risk of bias summary for randomized trials (RoB 2 tool)[59] 26
Supplement and Table 5. Grading of Recommendations, Assessment, Development, and Evaluations (GRADE)[60] 27
Supplement and References 28

Supplement eMethods 1. PRISMA Main Checklist[1]

Topic	No.	Item	Location where item is reported
**TITLE**			
**Title**	1	Identify the report as a systematic review	Pag.1
**ABSTRACT**			
**Abstract**	2	See the PRISMA 2020 for Abstracts checklist	Pag.2
**INTRODUCTION**			
**Rationale**	3	Describe the rationale for the review in the context of existing knowledge.	Pag.4
**Objectives**	4	Provide an explicit statement of the objective(s) or question(s) the review addresses.	Pag.4
**METHODS**			
**Eligibility criteria**	5	Specify the inclusion and exclusion criteria for the review and how studies were grouped for the syntheses.	Pag.5
**Information sources**	6	Specify all databases, registers, websites, organizations, reference lists and other sources searched or consulted to identify studies. Specify the date when each source was last searched or consulted.	Pag.5
**Search strategy**	7	Present the full search strategies for all databases, registers and websites, including any filters and limits used.	Supplement eMethods 3
**Selection process**	8	Specify the methods used to decide whether a study met the inclusion criteria of the review, including how many reviewers screened each record and each report retrieved, whether they worked independently, and if applicable, details of automation tools used in the process.	Pag.5
**Data collection process**	9	Specify the methods used to collect data from reports, including how many reviewers collected data from each report, whether they worked independently, any processes for obtaining or confirming data from study investigators, and if applicable, details of automation tools used in the process.	Pag.5
**Data items**	10a	List and define all outcomes for which data were sought. Specify whether all results that were compatible with each outcome domain in each study were sought (e.g. for all measures, time points, analyses), and if not, the methods used to decide which results to collect.	Pag.6
	10b	List and define all other variables for which data were sought (e.g. participant and intervention characteristics, funding sources). Describe any assumptions made about any missing or unclear information.	Pag.6

Topic	No.	Item	Location where item is reported
**Study risk of bias assessment**	11	Specify the methods used to assess risk of bias in the included studies, including details of the tool(s) used, how many reviewers assessed each study and whether they worked independently, and if applicable, details of automation tools used in the process.	Pag.6-7
**Effect measures**	12	Specify for each outcome the effect measure(s) (e.g. risk ratio, mean difference) used in the synthesis or presentation of results.	Pag. 7-8
**Synthesis methods**	13a	Describe the processes used to decide which studies were eligible for each synthesis (e.g. tabulating the study intervention characteristics and comparing against the planned groups for each synthesis (item 5)).	Pag. 7-8
	13b	Describe any methods required to prepare the data for presentation or synthesis, such as handling of missing summary statistics, or data conversions.	Pag. 7-8
	13c	Describe any methods used to tabulate or visually display results of individual studies and syntheses.	Pag. 7-8
	13d	Describe any methods used to synthesize results and provide a rationale for the choice(s). If meta-analysis was performed, describe the model(s), method(s) to identify the presence and extent of statistical heterogeneity, and software package(s) used.	Pag. 7-8
	13e	Describe any methods used to explore possible causes of heterogeneity among study results (e.g. subgroup analysis, meta-regression).	
	13f	Describe any sensitivity analyses conducted to assess robustness of the synthesized results.	Pag. 7-8
**Reporting bias assessment**	14	Describe any methods used to assess risk of bias due to missing results in a synthesis (arising from reporting biases).	NA
**Certainty assessment**	15	Describe any methods used to assess certainty (or confidence) in the body of evidence for an outcome.	NA
**RESULTS**			
**Study selection**	16a	Describe the results of the search and selection process, from the number of records identified in the search to the number of studies included in the review, ideally using a flow diagram.	Pag.8
	16b	Cite studies that might appear to meet the inclusion criteria, but which were excluded, and explain why they were excluded.	NA
**Study characteristics**	17	Cite each included study and present its characteristics.	Supplement eTable 1
**Risk of bias in studies**	18	Present assessments of risk of bias for each included study.	Supplement eResults 2
**Results of individual studies**	19	For all outcomes, present, for each study: (a) summary statistics for each group (where appropriate) and (b) an effect estimates and its precision (e.g. confidence/credible interval), ideally using structured tables or plots.	Pag.9-11

Topic	No.	Item	Location where item is reported
**Results of syntheses**	20a	For each synthesis, briefly summarize the characteristics and risk of bias among contributing studies.	Pag. 9-11
	20b	Present results of all statistical syntheses conducted. If meta-analysis was done, present for each the summary estimate and its precision (e.g. confidence/credible interval) and measures of statistical heterogeneity. If comparing groups, describe the direction of the effect.	Pag. 9-11
	20c	Present results of all investigations of possible causes of heterogeneity among study results.	Pag.11
	20d	Present results of all sensitivity analyses conducted to assess the robustness of the synthesized results.	Supplement eResults 1
**Reporting biases**	21	Present assessments of risk of bias due to missing results (arising from reporting biases) for each synthesis assessed.	Supplement eResults 2
**Certainty of evidence**	22	Present assessments of certainty (or confidence) in the body of evidence for each outcome assessed.	NA
**DISCUSSION**			
**Discussion**	23a	Provide a general interpretation of the results in the context of other evidence.	Pag. 12
	23b	Discuss any limitations of the evidence included in the review.	Pag. 14-15
	23c	Discuss any limitations of the review processes used.	Pag.14-15
	23d	Discuss implications of the results for practice, policy, and future research.	Pag.15
**OTHER INFORMATION**			
**Registration and protocol**	24a	Provide registration information for the review, including register name and registration number, or state that the review was not registered.	Pag. 5
	24b	Indicate where the review protocol can be accessed, or state that a protocol was not prepared.	Pag. 5
	24c	Describe and explain any amendments to information provided at registration or in the protocol.	Pag.5
**Support**	25	Describe sources of financial or non-financial support for the review, and the role of the funders or sponsors in the review.	Pag.17
**Competing interests**	26	Declare any competing interests of review authors.	Pag.17

Topic	No.	Item	Location where item is reported
**Availability of data, code and other materials**	27	Report which of the following are publicly available and where they can be found: template data collection forms; data extracted from included studies; data used for all analyses; analytic code; any other materials used in the review.	NA

Abbreviations: PRISMA, Preferred Reporting Items for Systematic Reviews and Meta-Analysis

Supplement eMethods 2. Details of the Search Strategy According to the Database

Database	Search strategy
**PubMed/ MEDLINE**	(“Myocardial Infarction”[mh] OR “myocardial infarction” OR MI OR “ST Elevation Myocardial Infarction”[mh] OR “ST-segment elevation myocardial infarction” OR STEMI OR STEACS OR “Non-ST Elevated Myocardial Infarction”[mh] OR “non–ST-segment elevation myocardial infarction” OR NSTEMI OR NSTEACS OR “Acute Coronary Syndrome”[mh] OR “acute coronary syndrome” OR ACS) AND (((blood OR “red blood cell*” RBC OR Erythrocytes[mh] OR erythrocyte* OR “packed red blood cell*” OR PRBC OR therapy) AND (transfusion*)) OR “Erythrocyte Transfusion”[mh] OR “Blood Transfusion”[mh]) AND (randomized controlled trial[pt] OR controlled clinical trial[pt] OR randomized[tiab] OR placebo[tiab] OR clinical trials as topic[mesh:noexp] OR randomly[tiab] OR trial[ti] OR Double-Blind Method [tiab] NOT (animals[mh] NOT humans [mh]))
**EMBASE**	('heart infarction'/exp OR 'heart infarction' OR 'myocardial infarction' OR mi OR 'st elevation myocardial infarction'/exp OR 'st segment elevation myocardial infarction' OR stemi OR steacs OR 'non st segment elevation myocardial infarction'/exp OR 'non st segment elevation myocardial infarction' OR nstemi OR nsteacs OR 'acute coronary syndrome'/exp OR 'acute coronary syndrome' OR acs) AND (((blood OR 'red blood cell*') AND rbc OR 'erythrocyte'/exp OR erythrocyte* OR 'packed red blood cell*' OR prbc OR therapy) AND transfusion* OR 'erythrocyte transfusion'/exp OR 'blood transfusion'/exp) AND ('randomized controlled trial':de OR random*:de,ab,ti OR rct:ti,ab,kw OR randomized:ti,ab,kw OR randomised:ti,ab,kw)
**COCHRANE**	(“myocardial infarction” OR MI OR “ST-segment elevation myocardial infarction” OR STEMI OR STEACS OR “non–ST-segment elevation myocardial infarction” OR NSTEMI OR NSTEACS OR “acute coronary syndrome” OR ACS) AND ((blood OR “red blood cell” RBC or erythrocyte OR “packed red blood cell” OR PRBC OR therapy) AND (transfusion)) AND (randomized controlled trial OR controlled clinical trial OR randomized OR placebo OR “clinical trials” OR randomly OR trial OR “Double-Blind Method”)
**ClinicalTrials.gov**	Condition/disease: ("Myocardial Infarction" OR "ST Elevation Myocardial Infarction" OR STEMI OR "ST-segment elevation myocardial infarction" OR NSTEMI OR STEACS OR NSTEACS OR "Non-ST Elevated Myocardial Infarction" OR "non–ST-segment elevation myocardial infarction" OR ACS OR "acute coronary syndrome")<br>Intervention/treatment: ((blood OR “red blood cell” RBC OR Erythrocytes OR erythrocyte OR “packed red blood cell” OR PRBC OR therapy) AND (transfusion))

Supplement eMethods 3. Definition of endpoints

Endpoint	CRIT 2011[2]	REALITY 2021[3]	MINT 2023[4]
**Cardiovascular Mortality**	-	Death resulting from an acute myocardial infarction, sudden cardiac death, heart failure, stroke, cardiovascular procedures, cardiovascular hemorrhage, and other cardiovascular causes	Death resulting from cardiovascular cause, as congestive heart failure and dysrhythmia
**Recurrent Myocardial Infarction**	Recurrent ischemic chest discomfort, new ischemic EKG changes, and CK-MB increase above the upper limit of normal and increased by ≥ 50% over the previous value	Rise and/or fall in cardiac biomarker (preferably troponin), associated with symptoms of ischemia, ischemic EKG changes, or imaging evidence of ischemia	Initial fall in the troponin and subsequent rise of at least 20% with additional evidence of MI (new EKG changes, imaging evidence, clinical history)
**Acute Heart Failure**	Patients with cardiogenic shock or patients with pulmonary vascular congestion, which physicians decided to treat with diuretics or vasoactive drug	Patients with new or worsening heart failure symptoms, as dyspnea, decreased exercise tolerance, fatigue; associated with objective evidence of heart failure (physical examination, laboratory, imaging)	Patients with new or worsening heart failure symptoms, as dyspnea, paroxysmal nocturnal dyspnea, orthopnea; associated with objective evidence of heart failure, receiving treatment specifically for exacerbation
**Stroke**	-	Acute episode of focal or global neurological dysfunction caused by central nervous system vascular injury because of hemorrhage or infarction	Acute episode of focal neurological dysfunction caused by central nervous system ischemia based on imaging or persistence of symptoms for more than 24 hours
**Unscheduled Revascularization**	-	Unscheduled coronary revascularization driven by recurrent acute ischemia	Unscheduled coronary revascularization driven by recurrent acute ischemia
**Acute Kidney Injury**	-	According to investigator judgment	Not described
**Infection**	-	Documented bacterial infection acquired at any time after the first transfusion	Documented pneumonia (radiographic abnormalities associated with symptoms, signs, or laboratory abnormalities), or blood stream infection (pathogen cultured from 1 or more blood cultures associated with fever, chills, or hypotension)

Endpoint	CRIT 2011[2]	REALITY 2021[3]	MINT 2023[4]
**Cardiovascular Mortality**	-	Death resulting from an acute myocardial infarction, sudden cardiac death, heart failure, stroke, cardiovascular procedures, cardiovascular hemorrhage, and other cardiovascular causes	Death resulting from cardiovascular cause, as congestive heart failure and dysrhythmia
**Severe Allergic Reaction**	-	According to investigator judgment	Not described
**Acute Lung Injury**	-	According to investigator judgment	Not described

Abbreviations: CK-MB, creatine kinase-myoglobin binding; EKG, electrocardiogram; MI, myocardial infarction

Supplement eMethods 4. Reproducible code on R software for calculations and graphics[5]

```
```{r}
if (!require(pacman)) install.packages(pacman)
p_load(tidyverse, meta, readxl, RTSA, cowplot)
```

```{r}
outcome = "All-Cause Mortality"

df <- read_excel("Coleta R - MI and anemia.xlsx", sheet = 1) |>
  mutate(study = case_when(study == "Cooper et al. 2011" ~ "CRIT 2011",
                     study == "MINT trial 2023" ~ "MINT 2023",
                     study == "Ducrocq et al. 2021" ~ "REALITY 2021") )
names(df)

ma <- metabin(events_treated, n_treated, events_control, n_control, study, df,
         method.tau = "REML", fixed = FALSE)
summary(ma)

pdf("forest-all_cause_mortality.pdf", 9, 3); ma |>
  forest(smlab = outcome, layout = "RevMan5", sortvar = TE,
       test.overall = TRUE, digits.TE = 2, digits.se = 2,
       label.left = "Favors Restrictive", label.right = "Favors Liberal",
       label.e = "Restrictive", label.c = "Liberal", subgroup.name = "",
       col.subgroup = "black", col.square = "#1a759f", p.col = "#1d3557",
       col.square.lines = NA_character_,); dev.off()

pdf("leave_one_out-all_cause_mortality.pdf", 7, 3); ma |>
  metainf() |>
  forest(smlab = outcome, layout = "RevMan5", sortvar = TE,
       test.overall = TRUE, digits.TE = 2, digits.se = 2,
       label.left = "Favors Restrictive", label.right = "Favors Liberal",
       label.e = "Restrictive", label.c = "Liberal", subgroup.name = "",
       col.subgroup = "black", col.square = "#1a759f", p.col = "#1d3557",
       col.square.lines = NA_character_,); dev.off()
```

```{r}
outcome = "Heart Failure"

df <- read_excel("Coleta R - MI and anemia.xlsx", sheet = 2) |>
  mutate(study = case_when(study == "Cooper et al. 2011" ~ "CRIT 2011",
                     study == "MINT trial 2023" ~ "MINT 2023",
                     study == "Ducrocq et al. 2021" ~ "REALITY 2021") )
names(df)

ma <- metabin(events_treated, n_treated, events_control, n_control, study, df,
         method.tau = "REML", fixed = FALSE)
summary(ma)

pdf("forest-heart_failure.pdf", 9, 3); ma |>
  forest(smlab = outcome, layout = "RevMan5", sortvar = TE,
       test.overall = TRUE, digits.TE = 2, digits.se = 2,
       label.left = "Favors Restrictive", label.right = "Favors Liberal",
       label.e = "Restrictive", label.c = "Liberal", subgroup.name = "",
       col.subgroup = "black", col.square = "#1a759f", p.col = "#1d3557",
       col.square.lines = NA_character_,); dev.off()

pdf("leave_one_out-heart_failure.pdf", 7, 3); ma |>
```
```

```
  metainf() |>
  forest(smlab = outcome, layout = "RevMan5", sortvar = TE,
         test.overall = TRUE, digits.TE = 2, digits.se = 2,
         label.left = "Favors Restrictive", label.right = "Favors Liberal",
         label.e = "Restrictive", label.c = "Liberal", subgroup.name = "",
         col.subgroup = "black", col.square = "#1a759f", p.col = "#1d3557",
         col.square.lines = NA_character_,); dev.off()

```

```
```{r}
outcome = "Myocardial Infarction"

df <- read_excel("Coleta R - MI and anemia.xlsx", sheet = 3) |>
  mutate(study = case_when(study == "Cooper et al. 2011" ~ "CRIT 2011",
                           study == "MINT trial 2023" ~ "MINT 2023",
                           study == "Ducrocq et al. 2021" ~ "REALITY 2021") )
names(df)

ma <- metabin(events_treated, n_treated, events_control, n_control, study, df,
              method.tau = "REML", fixed = FALSE)
summary(ma)

pdf("forest-myocardial_infarction.pdf", 9, 3); ma |>
  forest(smlab = outcome, layout = "RevMan5", sortvar = TE,
         test.overall = TRUE, digits.TE = 2, digits.se = 2,
         label.left = "Favors Restrictive", label.right = "Favors Liberal",
         label.e = "Restrictive", label.c = "Liberal", subgroup.name = "",
         col.subgroup = "black", col.square = "#1a759f", p.col = "#1d3557",
         col.square.lines = NA_character_,); dev.off()

pdf("leave_one_out-myocardial_infarction.pdf", 7, 3); ma |>
  metainf() |>
  forest(smlab = outcome, layout = "RevMan5", sortvar = TE,
         test.overall = TRUE, digits.TE = 2, digits.se = 2,
         label.left = "Favors Restrictive", label.right = "Favors Liberal",
         label.e = "Restrictive", label.c = "Liberal", subgroup.name = "",
         col.subgroup = "black", col.square = "#1a759f", p.col = "#1d3557",
         col.square.lines = NA_character_,); dev.off()

```

```
```{r}
outcome = "Cardiovascular Mortality"

df <- read_excel("Coleta R - MI and anemia.xlsx", sheet = 4) |>
  mutate(study = case_when(study == "Cooper et al. 2011" ~ "CRIT 2011",
                           study == "MINT trial 2023" ~ "MINT 2023",
                           study == "Ducrocq et al. 2021" ~ "REALITY 2021") )
names(df)

ma <- metabin(events_treated, n_treated, events_control, n_control, study, df,
              method.tau = "REML", fixed = FALSE)
summary(ma)

pdf("forest-cardiovascular_mortality.pdf", 9, 3); ma |>
  forest(smlab = outcome, layout = "RevMan5", sortvar = TE,
         test.overall = TRUE, digits.TE = 2, digits.se = 2,
         label.left = "Favors Restrictive", label.right = "Favors Liberal",
         label.e = "Restrictive", label.c = "Liberal", subgroup.name = "",
```

```
        col.subgroup = "black", col.square = "#1a759f", p.col = "#1d3557",
        col.square.lines = NA_character_,); dev.off()

pdf("leave_one_out-cardiovascular_mortality.pdf", 7, 3); ma |>
  metainf() |>
  forest(smlab = outcome, layout = "RevMan5", sortvar = TE,
        test.overall = TRUE, digits.TE = 2, digits.se = 2,
        label.left = "Favors Restrictive", label.right = "Favors Liberal",
        label.e = "Restrictive", label.c = "Liberal", subgroup.name = "",
        col.subgroup = "black", col.square = "#1a759f", p.col = "#1d3557",
        col.square.lines = NA_character_,); dev.off()

```

```{r}
outcome = "Stroke"

df <- read_excel("Coleta R - MI and anemia.xlsx", sheet = 5) |>
  mutate(study = case_when(study == "Cooper et al. 2011" ~ "CRIT 2011",
                      study == "MINT trial 2023" ~ "MINT 2023",
                      study == "Ducrocq et al. 2021" ~ "REALITY 2021") )
names(df)

ma <- metabin(events_treated, n_treated, events_control, n_control, study, df,
          method.tau = "REML", fixed = FALSE)
summary(ma)

pdf("forest-stroke.pdf", 9, 3); ma |>
  forest(smlab = outcome, layout = "RevMan5", sortvar = TE,
        test.overall = TRUE, digits.TE = 2, digits.se = 2,
        label.left = "Favors Restrictive", label.right = "Favors Liberal",
        label.e = "Restrictive", label.c = "Liberal", subgroup.name = "",
        col.subgroup = "black", col.square = "#1a759f", p.col = "#1d3557",
        col.square.lines = NA_character_,); dev.off()

pdf("leave_one_out-stroke.pdf", 7, 3); ma |>
  metainf() |>
  forest(smlab = outcome, layout = "RevMan5", sortvar = TE,
        test.overall = TRUE, digits.TE = 2, digits.se = 2,
        label.left = "Favors Restrictive", label.right = "Favors Liberal",
        label.e = "Restrictive", label.c = "Liberal", subgroup.name = "",
        col.subgroup = "black", col.square = "#1a759f", p.col = "#1d3557",
        col.square.lines = NA_character_,); dev.off()

```

```{r}
outcome = "Unscheduled Revascularization"

df <- read_excel("Coleta R - MI and anemia.xlsx", sheet = 6) |>
  mutate(study = case_when(study == "Cooper et al. 2011" ~ "CRIT 2011",
                      study == "MINT trial 2023" ~ "MINT 2023",
                      study == "Ducrocq et al. 2021" ~ "REALITY 2021") )
names(df)

ma <- metabin(events_treated, n_treated, events_control, n_control, study, df,
          method.tau = "REML", fixed = FALSE)
summary(ma)

pdf("forest-unscheduled_revascularization.pdf", 9, 3); ma |>
```

```
  forest(smlab = outcome, layout = "RevMan5", sortvar = TE,
        test.overall = TRUE, digits.TE = 2, digits.se = 2,
        label.left = "Favors Restrictive", label.right = "Favors Liberal",
        label.e = "Restrictive", label.c = "Liberal", subgroup.name = "",
        col.subgroup = "black", col.square = "#1a759f", p.col = "#1d3557",
        col.square.lines = NA_character_,); dev.off()

pdf("leave_one_out-unscheduled_revascularization.pdf", 7, 3); ma |>
  metainf() |>
  forest(smlab = outcome, layout = "RevMan5", sortvar = TE,
        test.overall = TRUE, digits.TE = 2, digits.se = 2,
        label.left = "Favors Restrictive", label.right = "Favors Liberal",
        label.e = "Restrictive", label.c = "Liberal", subgroup.name = "",
        col.subgroup = "black", col.square = "#1a759f", p.col = "#1d3557",
        col.square.lines = NA_character_,); dev.off()

```

```
```{r}
outcome = "Acute Kidney Injury"

df <- read_excel("Coleta R - MI and anemia.xlsx", sheet = 7) |>
  mutate(study = case_when(study == "Cooper et al. 2011" ~ "CRIT 2011",
                           study == "MINT trial 2023" ~ "MINT 2023",
                           study == "Ducrocq et al. 2021" ~ "REALITY 2021") )
names(df)

ma <- metabin(events_treated, n_treated, events_control, n_control, study, df,
              method.tau = "REML", fixed = FALSE)
summary(ma)

pdf("forest-acute_kidney_injury.pdf", 9, 3); ma |>
  forest(smlab = outcome, layout = "RevMan5", sortvar = TE,
        test.overall = TRUE, digits.TE = 2, digits.se = 2,
        label.left = "Favors Restrictive", label.right = "Favors Liberal",
        label.e = "Restrictive", label.c = "Liberal", subgroup.name = "",
        col.subgroup = "black", col.square = "#1a759f", p.col = "#1d3557",
        col.square.lines = NA_character_,); dev.off()

pdf("leave_one_out-acute_kidney_injury.pdf", 7, 3); ma |>
  metainf() |>
  forest(smlab = outcome, layout = "RevMan5", sortvar = TE,
        test.overall = TRUE, digits.TE = 2, digits.se = 2,
        label.left = "Favors Restrictive", label.right = "Favors Liberal",
        label.e = "Restrictive", label.c = "Liberal", subgroup.name = "",
        col.subgroup = "black", col.square = "#1a759f", p.col = "#1d3557",
        col.square.lines = NA_character_,); dev.off()

```

```{r}
outcome = "Severe Allergic Reaction"

df <- read_excel("Coleta R - MI and anemia.xlsx", sheet = 8) |>
  mutate(study = case_when(study == "Cooper et al. 2011" ~ "CRIT 2011",
                           study == "MINT trial 2023" ~ "MINT 2023",
                           study == "Ducrocq et al. 2021" ~ "REALITY 2021") )
names(df)

ma <- metabin(events_treated, n_treated, events_control, n_control, study, df,
```
```

```
            method.tau = "REML", fixed = FALSE)
summary(ma)

pdf("forest-severe_allergic_reaction.pdf", 9, 3); ma |>
  forest(smlab = outcome, layout = "RevMan5", sortvar = TE,
         test.overall = TRUE, digits.TE = 2, digits.se = 2,
         label.left = "Favors Restrictive", label.right = "Favors Liberal",
         label.e = "Restrictive", label.c = "Liberal", subgroup.name = "",
         col.subgroup = "black", col.square = "#1a759f", p.col = "#1d3557",
         col.square.lines = NA_character_,); dev.off()

pdf("leave_one_out-severe_allergic_reaction.pdf", 7, 3); ma |>
  metainf() |>
  forest(smlab = outcome, layout = "RevMan5", sortvar = TE,
         test.overall = TRUE, digits.TE = 2, digits.se = 2,
         label.left = "Favors Restrictive", label.right = "Favors Liberal",
         label.e = "Restrictive", label.c = "Liberal", subgroup.name = "",
         col.subgroup = "black", col.square = "#1a759f", p.col = "#1d3557",
         col.square.lines = NA_character_,); dev.off()

```

```{r}
outcome = "Infection"

df <- read_excel("Coleta R - MI and anemia.xlsx", sheet = 9) |>
  mutate(study = case_when(study == "Cooper et al. 2011" ~ "CRIT 2011",
                        study == "MINT trial 2023" ~ "MINT 2023",
                        study == "Ducrocq et al. 2021" ~ "REALITY 2021") )
names(df)

ma <- metabin(events_treated, n_treated, events_control, n_control, study, df,
            method.tau = "REML", fixed = FALSE)
summary(ma)

pdf("forest-infection.pdf", 9, 3); ma |>
  forest(smlab = outcome, layout = "RevMan5", sortvar = TE,
         test.overall = TRUE, digits.TE = 2, digits.se = 2,
         label.left = "Favors Restrictive", label.right = "Favors Liberal",
         label.e = "Restrictive", label.c = "Liberal", subgroup.name = "",
         col.subgroup = "black", col.square = "#1a759f", p.col = "#1d3557",
         col.square.lines = NA_character_,); dev.off()

pdf("leave_one_out-infection.pdf", 7, 3); ma |>
  metainf() |>
  forest(smlab = outcome, layout = "RevMan5", sortvar = TE,
         test.overall = TRUE, digits.TE = 2, digits.se = 2,
         label.left = "Favors Restrictive", label.right = "Favors Liberal",
         label.e = "Restrictive", label.c = "Liberal", subgroup.name = "",
         col.subgroup = "black", col.square = "#1a759f", p.col = "#1d3557",
         col.square.lines = NA_character_,); dev.off()

```

```{r}
outcome = "Acute Lung Injury"

df <- read_excel("Coleta R - MI and anemia.xlsx", sheet = 10) |>
  mutate(study = case_when(study == "Cooper et al. 2011" ~ "CRIT 2011",
                        study == "MINT trial 2023" ~ "MINT 2023",
```

```
                        study == "Ducrocq et al. 2021" ~ "REALITY 2021") )
names(df)

ma <- metabin(events_treated, n_treated, events_control, n_control, study, df,
            method.tau = "REML", fixed = FALSE)
summary(ma)

pdf("forest-acute_lung_injury.pdf", 9, 3); ma |>
  forest(smlab = outcome, layout = "RevMan5", sortvar = TE,
        test.overall = TRUE, digits.TE = 2, digits.se = 2,
        label.left = "Favors Restrictive", label.right = "Favors Liberal",
        label.e = "Restrictive", label.c = "Liberal", subgroup.name = "",
        col.subgroup = "black", col.square = "#1a759f", p.col = "#1d3557",
        col.square.lines = NA_character_,); dev.off()

pdf("leave_one_out-acute_lung_injury.pdf", 7, 3); ma |>
  metainf() |>
  forest(smlab = outcome, layout = "RevMan5", sortvar = TE,
        test.overall = TRUE, digits.TE = 2, digits.se = 2,
        label.left = "Favors Restrictive", label.right = "Favors Liberal",
        label.e = "Restrictive", label.c = "Liberal", subgroup.name = "",
        col.subgroup = "black", col.square = "#1a759f", p.col = "#1d3557",
        col.square.lines = NA_character_,); dev.off()

` ` `


` ` `{r}
outcome = "Units of Blood Transfused"

df <- read_excel("Coleta R - MI and anemia.xlsx", sheet = 11) |>
  mutate(study = case_when(study == "CRIT" ~ "CRIT 2011",
                        study == "MINT" ~ "MINT 2023",
                        study == "REALITY" ~ "REALITY 2021") ) |>
  mutate(subgroup = ifelse(study == "MINT 2023", "MINT", "Previous Trials"))
names(df)

ma <- metacont(n_liberal, mean_liberal, sd_liberal,
        n_restrictive, mean_restrictive, sd_restrictive,
        study, df, method.tau = "REML", fixed = FALSE, subgroup = subgroup,
        subgroup.name = "")
summary(ma)

pdf("forest-units-of-blood-transfused.pdf", 9, 5); ma |>
  forest(smlab = outcome, layout = "RevMan5", sortvar = -TE,
        test.overall = TRUE, digits.TE = 2, digits.se = 2,
        label.left = "Higher in Restrictive", label.right = "Higher in Liberal",
        label.e = "Liberal", label.c = "Restrictive", subgroup.name = "",
        col.subgroup = "black", col.square = "#1a759f", p.col = "#1d3557",
        col.square.lines = NA_character_); dev.off()

pdf("leave_one_out-units-of-blood-transfused.pdf", 7, 3); ma |>
  metainf() |>
  forest(smlab = outcome, layout = "RevMan5", sortvar = TE,
        test.overall = TRUE, digits.TE = 2, digits.se = 2,
        label.left = "Favors Restrictive", label.right = "Favors Liberal",
        label.e = "Restrictive", label.c = "Liberal", subgroup.name = "",
        col.subgroup = "black", col.square = "#1a759f", p.col = "#1d3557",
        col.square.lines = NA_character_,); dev.off()

` ` `
```

````
```{r}
df <- data.frame(
  study = c("MINT 2023", "CRIT 2011", "REALITY 2021"),
  md = c(1.8, 0.9, -0.1),
  md_ll = c(1.67, -0.07, -0.59),
  md_ul = c(1.93, 1.87, 0.39),
  rr = c(1/1.19, 1/1.75, 1/0.72),
  rr_ll = c(1/1.47, 1/17.95, 1/1.28),
  rr_ul = c(1/0.96, 1/1.17, 1/0.40),
  n = c(3504, 45, 668)
)

pdf("transfusion-vs-mortality.pdf", 6, 5); ggplot(df, aes(x = md, y = rr, color = log(n, 10))) +
  geom_smooth(color = alpha("#1d3557", 0.5), linetype = "dotted") +
  geom_point() +
  geom_errorbar(aes(xmin = md_ll, xmax = md_ul), width = 0.1) +
  geom_errorbar(aes(ymin = rr_ll, ymax = rr_ul), width = 0.1) +
  scale_color_gradient(low = alpha("#1d3557", 0.5), high = "#1d3557") +
  theme_cowplot() +
  theme(legend.position = "none",
        axis.title.x = element_text(size = 12.5, margin = margin(t = 10)),
        axis.title.y = element_text(size = 12.5, margin = margin(r = 10))) +
  labs(x = "Mean difference in units of blood transfused\n(liberal vs. restrictive groups)",
       y = "Relative mortality risk\n(liberal vs. restrictive groups)") +
  annotate("text", x = df$md, y = df$rr_ul + 0.15, label = df$study) +
  scale_x_continuous(breaks = seq(-4, 4, 0.5)) +
  scale_y_continuous(breaks = seq(-4, 4, 0.5)); dev.off()
``
````
````

Supplement eTable 1. Detailed reason for exclusion in full-text review

Study	DOI or PMID	Reason for exclusion
Yu 1995[6]	10.1097/00003246-199506000-00006	Different intervention (different cardiac output)
Hébert 1999[7]	10.1056/NEJM199902113400601	Different population (critical care patients)
Bellomo 2001[8]	10.5694/j.1326-5377.2001.tb143630.x	Not a randomized controlled trial
Hébert 2001[9]	10.1097/00003246-200102000-00001	Different population (critical care patients)
Blomqvist 2003[10]	14619040	Not a randomized controlled trial
Besarab, 2005[11]	10.1159/000090191	Not a randomized controlled trial
Al-Sarraf 2005[12]	10.1503/cmaj.1041736	Not a randomized controlled trial
Cooper 2005[13]	NCT00126334	Study protocol
Yang 2005[14]	10.1016/j.jacc.2005.06.072	Not a randomized controlled trial
Alexander 2008[15]	10.1016/j.ahj.2008.01.009	Not a randomized controlled trial
Jolicoeur 2009[16]	10.1093/eurheartj/ehp279	Different intervention (pexelizumab versus placebo for patients with STEMI treated with primary PCI)
Lettino 2011[17]	10.1714/643.7497	Not a randomized controlled trial
Shehata 2012[18]	10.1111/j.1537-2995.2011.03236.x	Different population (patients undergoing cardiac surgery)
Sardar 2013[19]	10.1016/j.ahj.2013.07.021	Not a randomized controlled trial
Dunn 2013[20]	23552808	Different population (patients with gastrointestinal bleeding)
Carson 2013[121]	10.1001/jamainternmed.2013.2855	Not a randomized controlled trial
O'Malley 2013[22]	10.1001/jamainternmed.2013.1786	Not a randomized controlled trial
Carson 2013[23]	10.1001/jama.2012.50429	Not a randomized controlled trial
Carson 2013[24]	10.1016/j.ahj.2013.03.001	Different population (includes patients with stable and unstable angina)
Hanna 2013[25]	10.1016/j.amjcard.2012.12.041	Not a randomized controlled trial
Song 2014[26]	10.1016/j.athoracsur.2013.12.025	Not a randomized controlled trial
Willett 2014[27]	10.1016/j.cger.2014.01.006	Not a randomized controlled trial
Du Pont-Thibodeau 2014[28]	10.1186/2110-5820-4-16	Not a randomized controlled trial
Nakamura 2014[29]	10.1186/cc13297	Different population (elderly patients undergoing elective cardiac surgery)

Yazer 2014[230]	10.1111/trf.12706	Not a randomized controlled trial
Rao 2014[31]	10.1016/j.jacc.2013.11.028	Not a randomized controlled trial
Murphy 2015[32]	10.1056/NEJMoa1403612	Different population (patients undergoing elective cardiac surgery)
Ang 2015[33]	10.1111/vox.12359	Not a randomized controlled trial
Roubinian 2015[34]	10.1136/ebmed-2015-110218	Not a randomized controlled trial
Memtsoudis 2015[35]	10.1136/bmj.h3153	Not a randomized controlled trial
van Boven 2015[36]	L605197782	Not a randomized controlled trial
Holroyd 2015[37]	10.15420/icr.2015.10.1.22	Not a randomized controlled trial
Holst 2016[38]	26836806	Different population (critical care patients)
Reeves 2016[39]	10.3310/hta20600	Different population (patients undergoing cardiac surgery)
Perrin 2016[40]	10.1093/eurheartj/ehw433	Not a randomized controlled trial
Stokes 2016[41]	10.1136/bmjopen-2016-011311	Different population (patients undergoing cardiac surgery)
McKean 2016[42]	10.1016/j.otc.2016.02.005	Not a randomized controlled trial
Vincent 2016[43]	26859155	Not a randomized controlled trial
Frank 2016[44]	10.1001/jamasurg.2015.3399	Not a randomized controlled trial
Mazer 2017[45]	0.1053/j.jvca.2017.10.036	Different population (patients undergoing cardiac surgery)
Mazer 2017[46]	10.1056/NEJMoa1711818	Different population (patients undergoing cardiac surgery)
Aubron 2017[47]	10.1111/voxs.12364	Not a randomized controlled trial
De Silva 2017[48]	10.1136/heartjnl-2015-307602	Not a randomized controlled trial
Perrin 2017[49]	L617530483	Not a randomized controlled trial
Dejam 2018[50]	NCT01504945	Trial registry record
Mazer 2018[51]	10.1056/NEJMoa1808561	Different population (patients undergoing cardiac surgery)
Meybohm 2019[52]	10.1186/s13063-019-3200-3	Different population (elderly patients undergoing elective noncardiac surgery)
Estcourt 2019[53]	10.1111/tme.12596	Different population (patients undergoing cardiac surgery)
Deharo 2020[54]	10.1016/j.ijcard.2020.06.020	Different intervention (heparin plus eptifibatide versus otamixaban)
Shah 2020[55]	10.1111/anae.14973	Not a randomized controlled trial

Jiang 2021[56]	10.7326/ACPJ202107200-077	Not a randomized controlled trial
Gonzalez-Juanatey 2022[57]	10.1161/CIRCULATIONAHA.121.057909	Not a randomized controlled trial
Galan 2022[58]	10.1016/j.medine.2020.07.003	Different population (patients undergoing cardiac surgery)

Abbreviations: PCI, percutaneous coronary intervention; STEMI ST-segment elevation

Supplement eTable 2. Definition of restrictive and liberal transfusion strategies

Transfusion strategy	CRIT 2011[2]	REALITY 2021[3]	MINT 2023[4]
**Liberal**	RBC transfusion when Ht decreased < 30% with goal to maintain a Ht from 30% to 33%	RBC transfusion when Hb ≤ 10g/dL with goal to maintain Hb of at least 11 g/dL	RBC transfusion after randomization and when Hb < 10g/dL, with goal to maintain Hb at 10 g/dL or above
**Restrictive**	RBC transfusion when Ht decreased < 24% with a goal to maintain Ht from 24% to 27%.	RBC transfusion when Hb decreased ≤ 8g/dL with goal to maintain Hb from 8 to 10 g/dL	RBC transfusion when Hb ≤ 7 or 8 g/dL

Abbreviations: Hb, hemoglobin; Ht, hematocrit; RBC, red blood cell

Supplement eTable 3. Clinical baseline characteristics of the included trials

RCT name	MINT 2023 (N = 3504)[4]	REALITY 2021 (N = 668)[3]	CRIT 2011 (N = 45)[2]
Randomized Controlled Trials Characteristics			
Study design	Multicenter (multinational), open-label, superiority, phase 3 RCT	Multicenter (binational), open-label, noninferiority, phase 3 RCT	Three-center, open-label, pilot RCT
Key inclusion criteria	Age ≥ 18 years<br>Type 1, 2, 4b, or 4c AMI<br>Hb < 10 g/dL	Age ≥ 18 years<br>AMI<br>Hb 7-10 g/dL	AMI<br>Ht ≤ 30% within 72h of symptom onset
Key exclusion criteria	Uncontrolled bleeding<br>On palliative treatment<br>Scheduled for cardiac surgery	SBP < 90 mmHg at randomization<br>Massive, ongoing, life-threatening bleeding<br>Type 4 AMI	Active, meaningful bleeding
Transfusion strategy groups (cutoffs for transfusion) — No. (%)	Restrictive (Hb 7 or 8 g/dL), 1749 (50)<br>Liberal (Hb <10 g/dL), 1755 (50)	Restrictive (Hb <8 g/dL), 342 (51)<br>Liberal (Hb <10 g/dL), 324 (49)	Conservative (Ht <24%), 24 (53)<br>Liberal (Ht <30%), 21 (47)
Primary outcome	Composite of myocardial infarction or death at 30 days	MACE (composite of all cause death, stroke, recurrent MI, or emergency revascularization) at 30 days	Composite of in-hospital death, recurrent MI, or new or worsening HF at 30 days
Demographic Characteristics			
Mean age, mean ± SD	Restrictive, 72.2 ± 11.5<br>Liberal, 72.1 ± 11.6	Restrictive, 77.95 ± 2.75<br>Liberal, 76.02 ± 2.59	Restrictive, 70.3 ± 14.3<br>Liberal, 76.4 ± 13.5
Female sex (%)	Restrictive, 44.3<br>Liberal, 46.7	Restrictive, 41.2<br>Liberal, 43.2	Restrictive, 46<br>Liberal, 52
Race (%)	White, 70.6<br>Latino, 8.1<br>Black, 12.6<br>Other, 7.0	White, 85.6<br>Other, 14.4	White, 68.0<br>Other, 32.0
Hypertension (%)	Restrictive, 84.5<br>Liberal, 85.4	Restrictive, 79.5<br>Liberal, 79.0	Restrictive, 75<br>Liberal, 91
Dyslipidemia (%)	Restrictive, 64.2<br>Liberal, 65.4	Restrictive, 55.3<br>Liberal, 62.0	Restrictive, 63<br>Liberal, 76

Diabetes (%)	Restrictive, 54.2<br>Liberal, 54.0	Restrictive, 51.5<br>Liberal, 48.8	Restrictive, 54<br>Liberal, 81
Tobacco smoking status (%)	Never, 40.2<br>Former, 43.2<br>Current, 16.6	Never, 47.7<br>Former, 36.9<br>Current, 15.4	Current, 22.2
Myocardial infarction (%)	Restrictive, 33.7<br>Liberal, 33.1	Restrictive, 35.4<br>Liberal, 36.7	NA
Percutaneous coronary intervention (%)	Restrictive, 35.6<br>Liberal, 32.9	Restrictive, 33.3<br>Liberal, 34.3	Restrictive, 25.0<br>Liberal, 24.0
Coronary artery bypass grafting (%)	Restrictive, 21.3<br>Liberal, 22.2	Restrictive, 12.9<br>Liberal, 13.0	Restrictive, 17.0<br>Liberal, 29.0
Acute Heart failure (%)	Restrictive, 30.1<br>Liberal, 30.7	Restrictive, 12.9<br>Liberal, 11.7	NA
Atrial Fibrillation (%)	Restrictive, 26.0<br>Liberal, 25.4	Restrictive, 15.8<br>Liberal, 20.1	NA
Killip class (%)	NA	I, 56.8<br>II, 26.7<br>III, 13.9<br>IV, 2.7	I, 60.0<br>II, 15.6<br>III, 6.7<br>IV, 17.8
Chronic anemia (%)	Restrictive, 42.0<br>Liberal, 43.2	Restrictive, 17.8<br>Liberal, 19.1	NA
Cancer (%)	Restrictive, 22.7<br>Liberal, 21.2	Restrictive, 19.5<br>Liberal, 19.1	NA
Chronic obstructive pulmonary disease (%)	COPD/asthma: Restrictive, 24.6<br>COPD/asthma: Liberal, 23.3	Restrictive, 9.9<br>Liberal, 12.3	NA
End-stage renal disease (%)	Restrictive, 45.6<br>Liberal, 46.2	Restrictive, 7.3<br>Liberal, 9.3	NA
Cerebrovascular disease (%)	Restrictive, 18.1<br>Liberal, 17.2	NA	NA
Index myocardial infarction			
NSTEMI (%)	Restrictive, 81.8<br>Liberal, 80.8	Restrictive, 68.4<br>Liberal, 71.3	Restrictive, 54<br>Liberal, 67
STEMI (%)	Restrictive, 18.2<br>Liberal, 19.2	Restrictive, 31.6<br>Liberal, 28.7	Restrictive, 46<br>Liberal, 33

Type 1 AMI (%)	Restrictive, 41.7<br>Liberal, 41.6	NA	NA
Type 2 AMI (%)	Restrictive, 55.3<br>Liberal, 56.3	NA	NA
Number of vessels with >50% obstruction (%)	0, 7.3<br>1, 27.0<br>2, 25.9<br>3, 39.8	NA	NA
Findings before randomization			
Left ventricular ejection fraction — mean ± SD	Restrictive, 47.3 ± 13.4<br>Liberal, 47.5 ± 13.7	NA	Restrictive, 39 ± 15<br>Liberal, 47 ± 13
Creatinine — median (Q1, Q3) or mean ± SD	Restrictive, 1.4 (0.9, 2.6)<br>Liberal, 1.4 (0.9, 2.5)	Restrictive, 1.3 (0.9, 2.0)<br>Liberal, 1.2 (0.9, 2.2)	Restrictive, 2.4 ± 2.3<br>Liberal, 2.9 ± 2.3
Hb — mean ± SD	Restrictive, 8.6 ± 0.8<br>Liberal, 8.6 ± 0.8	Restrictive, 9.0 ± 0.8<br>Liberal, 9.1 ± 0.8	Restrictive, 9.2 ± 0.8<br>Liberal, 9.0 ± 0.6
Ht — mean ± SD	NA	NA	Restrictive, 27.5 ± 2.4<br>Liberal, 26.9 ± 1.9
Hemoglobin level (g/dL) (%)	<8, 22.6<br>8 - <9, 38.3<br>9 - <10, 39.1	NA	NA
Active bleeding (%)	Restrictive, 39.7<br>Liberal, 39.9	Restrictive, 10.5<br>Liberal, 15.1	NA
No. of units of blood transfused — mean ± SD	Restrictive, 0.7 ± 1.6<br>Liberal, 2.5 ± 2.3	Restrictive, 2.9 ± 3.7<br>Liberal, 2.8 ± 2.7	Restrictive, 1.6 ± 2.0<br>Liberal, 2.5 ± 1.3
Number of red blood cell units transfused (%)			
0	Restrictive, 66.3<br>Liberal, 5.1	Restrictive, 66.1<br>Liberal, 0.0	NA
1	Restrictive, 18.4<br>Liberal, 30.7	Restrictive, 7.6<br>Liberal, 13.0	NA
2	Restrictive, 8.6<br>Liberal, 30.3	Restrictive, 18.0<br>Liberal, 39.8	NA
≥ 3	Restrictive, 6.8<br>Liberal, 33.9	Restrictive, 7.03<br>Liberal, 31.4	NA

Other transfusions (%)			
Fresh frozen plasma transfusion	NA	Restrictive, 0.9<br>Liberal, 2.2	NA
Platelet transfusion	NA	Restrictive, 1.2<br>Liberal, 1.2	NA

Abbreviations: AMI, acute myocardial infarction; Hb, hemoglobin; HF, heart failure; Ht, hematocrit; MACE, major cardiovascular event; MI, myocardial infarction; NSTEMI, non-ST-segment elevation myocardial infarction; SBP, systolic blood pressure; SD, standard deviation; STEMI, ST-segment elevation myocardial infarction.

Supplement eFigure 1. Leave-one-out analysis for all-cause mortality (A), heart failure (B), and recurrent myocardial infarction (C)[59]

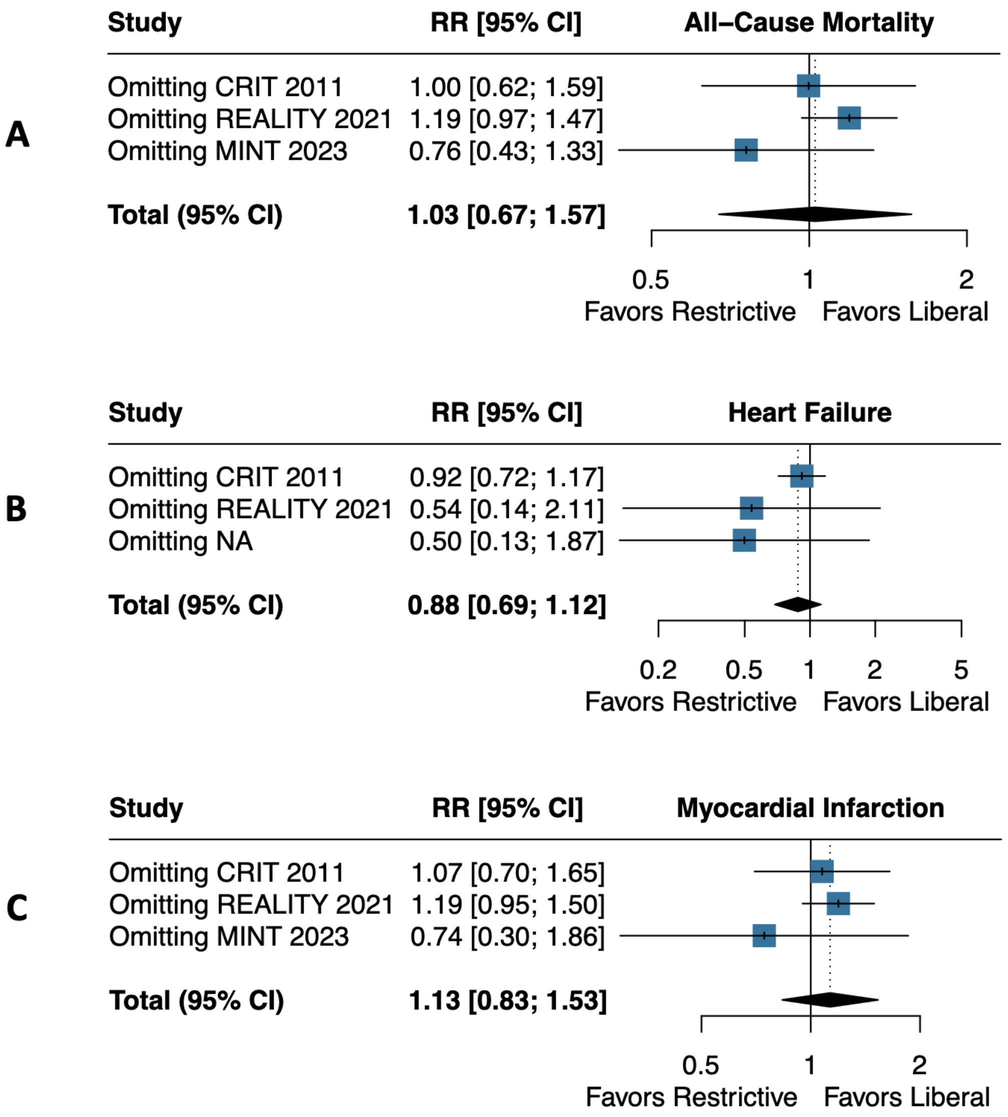

Supplement eTable 4. Risk of bias summary for randomized trials (RoB 2 tool)[60]

Risk of bias domains

Study	D1	D2	D3	D4	D5	Overall
CRIT 2011	+	+	+	+	+	+
REALITY 2021	+	+	+	+	+	+
MINT 2023	+	+	+	+	+	+

Domains:
D1: Bias arising from the randomization process.
D2: Bias due to deviations from intended intervention.
D3: Bias due to missing outcome data.
D4: Bias in measurement of the outcome.
D5: Bias in selection of the reported result.

Judgement
+ Low

Supplement eTable 5. Grading of Recommendations, Assessment, Development, and Evaluations (GRADE)[61]

Certainty assessment							Summary of findings				
Participants (studies) Follow-up	Risk of bias	Inconsistency	Indirectness	Imprecision	Publication bias	Overall certainty of evidence	Study event rates (%)		Relative effect (95% CI)	Anticipated absolute effects	
							With Liberal	With Restrictive		Risk with Liberal	Risk difference with Restrictive
**All-cause mortality**											
4215 (3 RCTs)	not serious	not serious	not serious	serious[a]	none	⨁⨁⨁◯ Moderate	172/2100 (8.2%)	194/2115 (9.2%)	**RR 1.03** (0.67 to 1.57)	82 per 1,000	**2 more per 1,000** (from 27 fewer to 47 more)
**Recurrent myocardial infarction**											
4215 (3 RCTs)	not serious	not serious	not serious	serious[a]	none	⨁⨁⨁◯ Moderate	136/2100 (6.5%)	157/2115 (7.4%)	**RR 1.13** (0.83 to 1.53)	65 per 1,000	**8 more per 1,000** (from 11 fewer to 34 more)
**Heart failure**											
4215 (3 RCTs)	not serious	not serious	not serious	serious[a]	none	⨁⨁⨁◯ Moderate	131/2100 (6.2%)	115/2115 (5.4%)	**RR 0.88** (0.69 to 1.12)	62 per 1,000	**7 fewer per 1,000** (from 19 fewer to 7 more)

CI: confidence interval; **RR:** risk ratio

Explanations

a. High imprecision due to broad confidence interval. Downgraded by one level for imprecision.

Supplement eReferences

1. Page MJ, McKenzie JE, Bossuyt PM, et al. The PRISMA 2020 statement: an updated guideline for reporting systematic reviews. *BMJ*. 2021;372. doi:10.1136/bmj.n71

2. Cooper HA, Rao SV, Greenberg, et al. Conservative versus liberal red cell transfusion in acute myocardial infarction (the CRIT Randomized Pilot Study). *Am J Cardiol*. 2011;108(8). doi:10.1016/j.amjcard.2011.06.014

3. Ducrocq G, Gonzalez-Juanatey JR, Puymirat E, et al. Effect of a Restrictive vs Liberal Blood Transfusion Strategy on Major Cardiovascular Events Among Patients With Acute Myocardial Infarction and Anemia: The REALITY Randomized Clinical Trial. *JAMA*. 2021;325(6). doi:10.1001/jama.2021.0135

4. Carson JL, Brooks MM, Hébert PC, et al. Restrictive or Liberal Transfusion Strategy in Myocardial Infarction and Anemia. *N Engl J Med*. Published online November 11, 2023. doi:10.1056/NEJMoa2307983

5. Harrer M, Cuijpers P, Furukawa TA, Ebert DD. *Doing Meta-Analysis with R: A Hands-On Guide*. CRC Press; 2021.

6. Yu M, Takanishi D, Myers SA, et al. Frequency of mortality and myocardial infarction during maximizing oxygen delivery: a prospective, randomized trial. *Crit Care Med*. 1995;23(6). doi:10.1097/00003246-199506000-00006

7. Hébert PC, Wells G, Blajchman MA, et al. A multicenter, randomized, controlled clinical trial of transfusion requirements in critical care. Transfusion Requirements in Critical Care Investigators, Canadian Critical Care Trials Group. *N Engl J Med*. 1999;340(6). doi:10.1056/NEJM199902113400601

8. Bellomo R. Will less liberal red-cell transfusion (with a lower haemoglobin threshold) still reduce rates of death and organ failure? *Med J Aust*. 2001;175(7). doi:10.5694/j.1326-5377.2001.tb143630.x

9. Hébert PC, Yetisir E, Martin C, et al. Is a low transfusion threshold safe in critically ill patients with cardiovascular diseases? *Crit Care Med*. 2001;29(2). doi:10.1097/00003246-200102000-00001

10. Blomqvist H, Sondell K. [Intensive care patients need blood transfusion--with limits. Risks must be weighed against potential benefit]. *Lakartidningen*. 2003;100(42). Accessed December 31, 2023. https://pubmed.ncbi.nlm.nih.gov/14619040/

11. Besarab A, Soman S. Anemia management in chronic heart failure: lessons learnt from chronic kidney disease. *Kidney Blood Press Res*. 2005;28(5-6). doi:10.1159/000090191

12. Al-Sarraf A, Fowler RA. Is blood transfusion harmful in patients with acute coronary syndromes? *CMAJ*. 2005;172(2). doi:10.1503/cmaj.1041736

13. Cooper HA. Conservative Versus Liberal Red Cell Transfusion in Myocardial Infarction Trial: the CRIT Pilot. Cochrane Library. Published 2005. https://www.cochranelibrary.com/central/doi/10.1002/central/CN-01508944/full

14. Yang X, Alexander KP, Chen AY, et al. The implications of blood transfusions for patients with non-ST-segment elevation acute coronary syndromes: results from the CRUSADE National Quality Improvement Initiative. *J Am Coll Cardiol*. 2005;46(8). doi:10.1016/j.jacc.2005.06.072

15. Alexander KP, Chen AY, Wang TY, et al. Transfusion practice and outcomes in non-ST-segment elevation acute coronary syndromes. *Am Heart J*. 2008;155(6). doi:10.1016/j.ahj.2008.01.009

16. Jolicoeur EM, O'Neill WW, Hellkamp A, et al. Transfusion and mortality in patients with ST-segment elevation myocardial infarction treated with primary percutaneous coronary intervention. *Eur Heart J*. 2009;30(21). doi:10.1093/eurheartj/ehp279

17. Lettino M, Toschi V. [Impact of anemia and its treatment in patients with acute coronary syndromes]. *G Ital Cardiol* . 2011;12(5). doi:10.1714/643.7497

18. Shehata N, Burns LA, Nathan H, et al. A randomized controlled pilot study of adherence to transfusion strategies in cardiac surgery. *Transfusion* . 2012;52(1). doi:10.1111/j.1537-2995.2011.03236.x

19. Sardar P, Nairooz R, Dutu L, Pastori LJ. Liberal versus restrictive transfusion strategy for patients with coronary artery disease. *Am Heart J*. 2013;166(4). doi:10.1016/j.ahj.2013.07.021

20. Dunn A. ACP Journal Club. A restrictive transfusion strategy reduced 45-day mortality in patients with acute upper GI bleeding. *Ann Intern Med*. 2013;158(6). doi:10.7326/0003-4819-158-6-201303190-02006

21. Carson JL, Hébert PC. Here we go again--blood transfusion kills patients?: comment on "Association of blood transfusion with increased mortality in myocardial infarction: a meta-analysis and diversity-adjusted study sequential analysis." *JAMA Intern Med*. 2013;173(2). doi:10.1001/jamainternmed.2013.2855

22. O'Malley PG. Less transfusion is likely more in acute myocardial infarction: comment on "Association of blood transfusion with increased mortality in myocardial infarction: a meta-analysis and diversity-adjusted study sequential analysis." *JAMA Intern Med*. 2013;173(2). doi:10.1001/jamainternmed.2013.1786

23. Carson JL, Carless PA, Hébert PC. Outcomes using lower vs higher hemoglobin thresholds for red blood cell transfusion. *JAMA*. 2013;309(1). doi:10.1001/jama.2012.50429

24. Carson JL, Brooks MM, Abbott JD, et al. Liberal versus restrictive transfusion thresholds for patients with symptomatic coronary artery disease. *Am Heart J*. 2013;165(6). doi:10.1016/j.ahj.2013.03.001

25. Hanna EB, Alexander KP, Chen AY, Roe MT, Funk M, Saucedo JF. Characteristics and in-hospital outcomes of patients with non-ST-segment elevation myocardial infarction undergoing an invasive strategy according to hemoglobin levels. *Am J Cardiol*. 2013;111(8). doi:10.1016/j.amjcard.2012.12.041

26. Song HK, von Heymann C, Jespersen CM, et al. Safe application of a restrictive transfusion protocol in moderate-risk patients undergoing cardiac operations. *Ann Thorac Surg*. 2014;97(5). doi:10.1016/j.athoracsur.2013.12.025

27. Willett LR, Carson JL. Management of postoperative complications: anemia. *Clin Geriatr Med*. 2014;30(2). doi:10.1016/j.cger.2014.01.006

28. Du Pont-Thibodeau G, Harrington K, Lacroix J. Anemia and red blood cell transfusion in critically ill cardiac patients. *Ann Intensive Care*. 2014;4. doi:10.1186/2110-5820-4-16

29. Nakamura R, Vincent JL, Fukushima J, et al. A liberal strategy of red blood cell transfusion reduces cardiovascular complications in older patients undergoing cardiac surgery. *Crit Care*. 2014;18(Suppl 1):P107.

30. Yazer MH, Triulzi DJ. Things aren't always as they seem: what the randomized trials of red blood cell transfusion tell us about adverse outcomes. *Transfusion* . 2014;54(12). doi:10.1111/trf.12706

31. Rao SV, Sherwood MW. Isn't it about time we learned how to use blood transfusion in patients with ischemic heart disease? *J Am Coll Cardiol*. 2014;63(13). doi:10.1016/j.jacc.2013.11.028

32. Murphy GJ, Pike K, Rogers CA, et al. Liberal or restrictive transfusion after cardiac surgery. *N Engl J Med*. 2015;372(11). doi:10.1056/NEJMoa1403612

33. Abstracts of the 26th Regional Congress of the International Society of Blood Transfusion in conjunction with the 6(th) Annual Conference of The Indonesian Society of Transfusion Medicine, Bali, Indonesia, November 14-16, 2015. *Vox Sang*. 2015;109 Suppl 2(Suppl 2). doi:10.1111/vox.12359

34. Roubinian NH, Carson JL. Restrictive red blood cell transfusion strategies appear safe in most clinical settings. *Evid Based Med*. 2015;20(5). doi:10.1136/ebmed-2015-110218

35. Memtsoudis SG. Perioperative blood transfusions. *BMJ*. 2015;350. doi:10.1136/bmj.h3153

36. [No title]. Accessed December 31, 2023. https://www.embase.com/records?subaction=viewrecord&id=L605197782

37. Holroyd EW, Mustafa AH, Khoo CW, et al. Major Bleeding and Adverse Outcome following Percutaneous Coronary Intervention. *Interventional cardiology (London, England)*. 2015;10(1). doi:10.15420/icr.2015.10.1.22

38. Holst LB. Benefits and harms of red blood cell transfusions in patients with septic shock in the intensive care unit. *Dan Med J*. 2016;63(2). Accessed December 31, 2023. https://pubmed.ncbi.nlm.nih.gov/26836806/

39. Reeves BC, Pike K, Rogers CA, et al. A multicentre randomised controlled trial of Transfusion Indication Threshold Reduction on transfusion rates, morbidity and health-care resource use following cardiac surgery (TITRe2). *Health Technol Assess*. 2016;20(60). doi:10.3310/hta20600

40. Perrin S., Putot A., Beer F., Maza M., Cottin Y., Zeller M., Manckoundia P. Effects of blood transfusion in elderly with acute myocardial infarction on one-year mortality. *European Heart Journal*. 2016;37. Accessed December 31, 2023. https://www.embase.com/records?subaction=viewrecord&id=L612285144

41. Stokes EA, Wordsworth S, Bargo D, et al. Are lower levels of red blood cell transfusion more cost-effective than liberal levels after cardiac surgery? Findings from the TITRe2 randomised controlled trial. *BMJ Open*. 2016;6(8). doi:10.1136/bmjopen-2016-011311

42. McKean E. Quality Control Approach to Anticoagulants and Transfusion. *Otolaryngol Clin North Am*. 2016;49(3). doi:10.1016/j.otc.2016.02.005

43. Vincent JL, VAN DER Linden P. Restrictive versus more liberal blood transfusions? The answer is in your heart. *Minerva Anestesiol*. 2016;82(5). Accessed December 31, 2023. https://pubmed.ncbi.nlm.nih.gov/26859155/

44. Frank SM, Ejaz A, Pawlik TM. Optimal Transfusion Trigger in Surgical Patients With Coronary Artery Disease. *JAMA Surg*. 2016;151(2). doi:10.1001/jamasurg.2015.3399

45. Shehata N, Whitlock R, Fergusson DA, et al. Transfusion Requirements in Cardiac Surgery III (TRICS III): Study Design of a Randomized Controlled Trial. *J Cardiothorac Vasc Anesth*. 2018;32(1). doi:10.1053/j.jvca.2017.10.036

46. Mazer CD, Whitlock RP, Fergusson DA, et al. Restrictive or Liberal Red-Cell Transfusion for Cardiac Surgery. *N Engl J Med*. 2017;377(22). doi:10.1056/NEJMoa1711818

47. Aubron C. Making transfusion decisions in critical care. *ISBT Sci Ser*. 2017;12(4):429-434.

48. De Silva K, Myat A, Cotton J, James S, Gershlick A, Stone GW. Bleeding associated with the management of acute coronary syndromes. *Heart*. 2017;103(7). doi:10.1136/heartjnl-2015-307602

49. Perrin S., Putot A., Beer J.C., Maza M., Cottin Y., Zeller M., Manckoundia P. Effect of blood transfusion in elderly patients with acute myocardial infarction on one-year mortality. *Archives of Cardiovascular Diseases Supplements*. 2017;9(1):121-122.

50. Dejam A. CTG Labs - NCBI. ClinicalTrials.gov. Published 2018. https://clinicaltrials.gov/study/NCT01504945

51. Mazer CD, Whitlock RP, Fergusson DA, et al. Six-Month Outcomes after Restrictive or Liberal Transfusion for Cardiac Surgery. *N Engl J Med*. 2018;379(13). doi:10.1056/NEJMoa1808561

52. Meybohm P, Lindau S, Treskatsch S, et al. Liberal transfusion strategy to prevent mortality and anaemia-associated, ischaemic events in elderly non-cardiac surgical patients - the study design of the LIBERAL-

Trial. *Trials*. 2019;20(1). doi:10.1186/s13063-019-3200-3

53. Estcourt LJ, Roberts DJ. Six-month outcomes after restrictive or liberal transfusion for cardiac surgery (TRICS III trial). *Transfus Med*. 2019;29(2):77-79. doi:10.1111/tme.12596

54. Deharo P, Ducrocq G, Bode C, et al. Blood transfusion and ischaemic outcomes according to anemia and bleeding in patients with non-ST-segment elevation acute coronary syndromes: Insights from the TAO randomized clinical trial. *Int J Cardiol*. 2020;318. doi:10.1016/j.ijcard.2020.06.020

55. Shah A, Stanworth SJ, Docherty AB. Restrictive blood transfusion - is less really more? *Anaesthesia*. 2020;75(4). doi:10.1111/anae.14973

56. Jiang D, Garcia D. In patients with AMI and anemia, a restrictive vs. liberal blood transfusion strategy was noninferior for 30-d MACE. *Ann Intern Med*. 2021;174(7). doi:10.7326/ACPJ202107200-077

57. Gonzalez-Juanatey JR, Lemesle G, Puymirat E, et al. One-Year Major Cardiovascular Events After Restrictive Versus Liberal Blood Transfusion Strategy in Patients With Acute Myocardial Infarction and Anemia: The REALITY Randomized Trial. *Circulation*. 2022;145(6). doi:10.1161/CIRCULATIONAHA.121.057909

58. Galan J, Mateo E, Carmona P, Gajate L, Mazer CD, Martinez-Zapata MJ. Restrictive or liberal transfusion for cardiac surgery: Spanish results of a randomized multicenter international parallel open-label clinical trial. *Med Intensiva*. 2022;46(1). doi:10.1016/j.medine.2020.07.003

59. Willis BH, Riley RD. Measuring the statistical validity of summary meta-analysis and meta-regression results for use in clinical practice. *Stat Med*. 2017;36(21). doi:10.1002/sim.7372

60. Sterne JAC, Savović J, Page MJ, et al. RoB 2: a revised tool for assessing risk of bias in randomised trials. *BMJ*. 2019;366. doi:10.1136/bmj.l4898

61. GRADE handbook. Accessed December 31, 2023. http://guidelinedevelopment.org/handbook
