# Supplementary material for: Estratégias de Transfusão Restritiva versus Liberal em Infarto Agudo do Miocárdio e Anemia: Metanálise e Análise Sequencial de Ensaios Clínicos
Source: Arq Bras Cardiol. 2024 Oct 9;121(9):e20240158. [Article in Portuguese] doi: 10.36660/abc.20240158 (PMC12092027; doi:10.36660/abc.20240158)
Supplement: Translation

# Translation: Restrictive versus Liberal Transfusion Strategies in Acute Myocardial Infarction and Anemia: A Meta-Analysis and Trial Sequential Analysis

**DOI:** 10.36660/abc.20240158i

**Authors:** Ronaldo C. Fabiano, Lara Melo, Alleh Nogueira, Douglas M. Gewehr, Giuliano Generoso, Rhanderson Cardoso, Marcio S. Bittencourt

**Affiliations:** 1 Pittsburgh PA USA Department of Internal Medicine, University of Pittsburgh Medical Center (UPMC), Pittsburgh, PA – USA; 2 Farmington CT USA Department of Internal Medicine, University of Connecticut, Farmington, CT – USA; 3 Salvador BA Brazil Escola Bahiana de Medicina e Saúde Pública, Salvador, BA – Brazil; 4 Curitiba PR Brazil Instituto do Coração, Curitiba, PR – Brazil; 5 São Paulo SP Brazil Centro de Pesquisa Clínica e Epidemiológica - Universidade de São Paulo, São Paulo, SP – Brazil; 6 Boston MA USA Division of Cardiovascular Medicine, Brigham and Women's Hospital, Harvard Medical School, Boston, MA – USA; 7 Pittsburgh PA USA Department of Cardiology, University of Pittsburgh Medical Center (UPMC), Pittsburgh, PA – USA

**Keywords:** Meta-Analysis, Myocardial Infarction, Anemia, Blood Transfusion

## Abstract

**Background:**

The optimal transfusion strategy in acute myocardial infarction (AMI)-associated anemia remains uncertain.

**Objectives:**

To compare all-cause mortality between liberal versus restrictive transfusion strategies in patients with AMI-associated anemia, using a meta-analytic approach.

**Methods:**

Pubmed, Embase, and ClinicalTrials.gov were systematically searched for randomized controlled trials (RCTs) comparing liberal and restrictive transfusion strategies in AMI-associated anemia. Random-effects meta-analysis and trial sequential analysis (TSA) were conducted to compare blood use, efficacy, and safety endpoints. The p-values were 2-sided with an α of 0.05.

**Results:**

In a pooled analysis involving 4,217 participants from three RCTs followed-up for 30 days, no statistically significant differences emerged between restrictive and liberal strategies in all-cause mortality (RR 1.03; 95% CI 0.67–1.57; p=0.90) and other efficacy endpoints (recurrent AMI, unscheduled revascularization, acute heart failure, stroke, and acute kidney injury), as well as in safety endpoints including allergic reactions, infection, and acute lung injury. TSA did not reach futility boundaries. In patients assigned to restrictive strategy, substantial differences in transfusion use were observed across RCTs, correlating with mortality rates, and likely accounting for between-study heterogeneity in treatment effects.

**Conclusions:**

In patients with AMI-associated anemia, there is no clear superiority between liberal and restrictive transfusion strategies in all-cause mortality or other major outcomes in 30 days. However, the heterogeneity observed in blood use between the restrictive groups likely explains variable findings across RCTs.

## Introduction

The optimal threshold for red blood cell (RBC) transfusion in patients with acute myocardial infarction (AMI) and anemia remains undefined.^1^Growing evidence suggests no statistical distinction in 30-day mortality or major clinical outcomes between restrictive and liberal transfusion strategies in a range of diverse conditions.^1 – 11^

A restrictive transfusion strategy is generally associated with a substantial reduction in RBC transfusions across various clinical scenarios.^1^Nevertheless, there is a theoretical potential clinical benefit of a liberal transfusion strategy, aiming for higher hemoglobin (Hb) levels to increase oxygen availability in patients with AMI and anemia. Two prior randomized controlled trials (RCTs) comparing restrictive and liberal transfusion thresholds in patients with AMI and anemia revealed superiority for a primary composite outcome (in-hospital death, recurrent myocardial infarction, or new or worsening heart failure);^12^and a non-inferiority for a major adverse cardiovascular event (all-cause death, stroke, recurrent myocardial infarction, or emergency revascularization)^6^in 30-day between the two strategies.^6 , 12^

The most recent and largest RCT to date, involving 3504 patients suggested a trend towards lower mortality in the liberal group.^13^Given the uncertainty of optimal transfusion strategy in patients with AMI and anemia and the conflicting findings suggested by this recent largest trial,^13^we conducted a systematic review and meta-analysis comparing liberal versus restrictive transfusion strategies in this population ( Central Illustration ).

## Methods

This systematic review and meta-analysis was performed and reported following the Cochrane Collaboration Handbook for Systematic Reviews of Interventions and the Preferred Reporting Items for Systematic Reviews and Meta-Analysis (PRISMA) Statement guidelines (Supplementary material and Methods 1).^14 , 15^The meta-analysis protocol was prospectively registered at the International Prospective Register of Systematic Reviews (PROSPERO; CRD42023484239).^16^

### Data source and search strategy

We systematically searched PubMed (MEDLINE), Embase, Cochrane, and ClincalTrials.gov from database inception to November 16, 2023, and without language restrictions. The search terms included "myocardial infarction", "acute coronary syndrome", and "blood transfusion". The complete search strategy for each database is provided in Supplementary material and Methods 2. After removing duplicates, two authors (L.M. and R.F.) screened titles and abstracts and independently assessed full-text articles for inclusion based on prespecified criteria. Discrepancies were resolved in a panel discussion with a third author (A.N.). Moreover, we used backward snowballing (i.e., review of references) to identify relevant references from articles identified in the original search.^17^

### Eligibility criteria

We considered studies eligible for inclusion if they (1) were RCTs; (2) enrolled adult patients (≥ 18 years of age) with ST-segment elevation (STEMI) or non-ST-segment elevation myocardial infarction (NSTEMI) and anemia (Hb ≤10 g or hematocrit Ht ≤30%); (3) compared restricted versus liberal blood transfusion strategies; and (4) presented data regarding any endpoints of interest. Exclusion criteria were: (1) studies including patients with stable or unstable angina without stratifying data for those with AMI; (2) myocardial infarction (MI) occurring after coronary artery bypass grafting or percutaneous coronary intervention; (3) patients receiving palliative treatment; or (4) no data reported for any outcomes of interest.

### Data extraction

Two authors (L.M. and R.F.) independently extracted the data from each study using a standardized form including: authors, enrollment period, study publication year, inclusion and exclusion criteria, sample size, follow-up period, transfusion strategies, MI and anemia assessments, baseline patient characteristics, baseline patients’ medications, endpoint data – total number of patients and number of events (binary endpoints), and endpoint definitions. Disagreements were resolved in a panel discussion with a third author (A.N.).

### Endpoints

Our prespecified primary endpoint was all-cause mortality. The secondary efficacy endpoints included (1) cardiovascular mortality; (2) recurrent MI; (3) acute heart failure (HF); (4) stroke; (5) unscheduled revascularization; and (6) acute kidney injury. Our safety endpoints were (1) acute lung injury; (2) infection; and (3) severe allergic reaction. We compared differences in blood transfusion between studies and intervention groups. Detailed endpoint definitions for each included study are provided in Supplementary material and Methods 3.

### Quality assessment and risk of bias

Two independent authors (L.M. and R.F.) assessed the risk of bias in the included RCTs using Cochrane's Risk of Bias (RoB 2) tool for assessing the risk of bias in randomized studies for the primary and secondary endpoints considering intention-to-treat groups.^17^In the RoB 2 evaluation, each trial was scored as high risk, low risk, or some concerns in each of the five domains: selection, performance, detection, attrition, and reporting biases for the primary and secondary endpoints. The GRADE (Grading of Recommendations, Assessment, Development, and Evaluations) assessments of evidence quality was performed according to the GRADE handbook, and the summary of findings was compiled independently by the two authors (L.M. and R.F.) using the GRADEpro/GDT software.^18 , 19^The five GRADE domains (risk of bias, inconsistency, indirections, imprecision, and publication bias) were used to categorize the level of certainty as high, moderate, low, or very low. The GRADE assessment was performed for all outcomes reported by the three included trials. Disagreements were resolved in a panel discussion with a third author (A.N.). Egger's test was not performed given the small number of included studies (n < 10), as recommended by Cochrane.^14^

### Statistical analysis

The statistical analyses were conducted in line with Cochrane recommendations.^14^To accommodate anticipated methodological and clinical heterogeneities across studies, treatment effect estimates were pooled using the Mantel-Haenszel random-effects model. Due to the limited number of studies, the restricted maximum likelihood estimator was used to calculate heterogeneity variance τ^2^.^14^Binary and continuous endpoints were summarized using risk ratios (RR) and mean differences (MD), respectively, along with their respective 95% confidence intervals (CI). Treatment effects were two-tailed and considered statistically significant at *p* <0.05. We assessed heterogeneity with Cochrane's Q statistic and Higgins and Thompson's I^2^statistic, with p ≤ 0.10 indicating statistical significance.^20^Leave-one-out sensitivity analyses were also conducted to ensure the robustness of our findings. We used R version 4.2.2 and the extension package "meta" for all calculations and graphics.^21^The full reproducible R code is available in Supplementary material and Methods 4.

To better assess potential type 1 and type 2 errors, we performed a trial sequential analysis (TSA) for all-cause mortality.^22^We used a random-effects model with 95% CI, an information axis with sample size, type 1 error with 5% two-sided boundary, and power of 80%. The adjustment of the thresholds for the Z score was based on the O'Brien–Fleming alpha spending function. TSA was performed using the TSA program version 0.9.5.10 beta (Copenhagen Trial Unit, Centre for Clinical Intervention Research, Rigshospitalet, Copenhagen, Denmark).^23^

## Results

### Study selection and baseline characteristics

The systematic search yielded 4,187 articles and abstracts ( Figure 1 ). After removing duplicates and studies that met the exclusion criteria based on title and abstract review, 56 articles were retrieved and reviewed in full for possible inclusion. Finally, four studies met the inclusion criteria and were analyzed, but one study was removed due to the absence of stratified data for the AMI patients, as this study also included patients without AMI.^24^The reasons for exclusions in the full-text review are detailed in Supplementary material eTable 1 . We included 4,217 patients, with 2,115 (50.2%) assigned to restrictive blood transfusion and 2,100 (49.8%) assigned to liberal blood transfusion strategy. Mean age of patients was 72.8 years (range, 72.1-77.0 years), and 45% (range, 42.2-48.0%) were female ( Table 1 ). In all three studies, the outcomes of interest were assessed at 30 days. Definitions of restrictive and liberal transfusion strategies in each study are detailed in Supplementary material eTable 2 . Supplementary material eTable 3 summarize the clinical baseline characteristics of the included patients.

**Figure 1:** Study screening and selection.

**Table 1:** Baseline characteristics of included studies and patients

Study Characteristics		MINT, 2023 (N = 3504)		REALITY, 2021 (N = 668)		CRIT, 2011 (N = 45)	
Key inclusion criteria		Age ≥ 18 years AMI Hb <10 g/dL		Age ≥ 18 years AMI Hb 7-10 g/dL		AMI Ht ≤ 30% within 72 hours of symptom onset	
		Restrictive (N =1749)	Liberal (N =1755)	Restrictive (N =342)	Liberal (N =324)	Conservative (N =24)	Liberal (N =21)
Transfusion strategy, (%)		Hb 7 or 8 g/dL, (50)	Hb <10 g/dL, (50)	Hb <8 g/dL, (51)	Hb <10 g/dL, (49)	Ht <24%, (53)	Ht <30%, (47)
Age in years, mean ± SD		72.2 ±11.5	72.1 ±11.6	78 (69-85)	76 (69-84)	70.3 ±14.3	76.4 ±13.5
Female sex, No. (%)		774 (44.3)	819 (46.7)	141 (41.2)	140 (43.2)	11 (46)	11 (52)
Hypertension, No. (%)		1478 (84.5)	1498 (85.4)	272 (79.5)	256 (79.0)	18 (75)	19 (91)
Dyslipidemia, No. (%)		1123 (64.2)	1147 (65.4)	189 (55.3)	201 (62.0)	15 (63)	16 (76)
Diabetes, No. (%)		948 (54.2)	948 (54.0)	176 (51.5)	158 (48.8)	13 (54)	17 (81)
Current tobacco smoking, No. (%)		273 (16.6)	275 (16.6)	51 (16.1)	41 (14.0)	8 (33)	2 (10)
**History before index, No. (%)**							
	MI	589 (33.7)	549 (33.1)	121 (35.4)	119 (36.7)	NA	NA
	PCI	623 (35.6)	577 (32.9)	114 (33.3)	111 (34.3)	6 (25)	5 (24)
	CABG	372 (21.3)	390 (22.2)	44 (12.9)	42 (13.0)	4 (17)	6 (29)
	Acute Heart failure	527 (30.1)	539 (30.7)	44 (12.9)	38 (11.7)	NA	NA
	Chronic anemia	735 (42.0)	758 (43.2)	61 (17.8)	62 (19.1)	NA	NA
	Cancer	397 (22.7)	372 (21.2)	67 (19.5)	62 (19.1)	NA	NA
	ESRD	797 (45.6)	810 (46.2)	25 (7.3)	30 (9.3)	NA	NA
**Index AMI, No. (%)**							
	NSTEMI	1430 (81.8)	1418 (80.8)	234 (68.4)	231 (71.3)	13 (54)	14 (67)
	STEMI	319 (18.2)	337 (19.2)	108 (31.6)	93 (28.7)	11 (46)	7 (33)
**Findings before randomization**							
LVEF %, mean ± SD		47.3 ±13.4	47.5 ±13.7	NA	NA	39 ±15	47 ±13
Creatinine, median (Q1, Q3) or mean ± SD		1.4 (0.9, 2.6)	1.4 (0.9, 2.5)	1.3 (0.9, 2.0)	1.2 (0.9, 2.2)	2.4 ± 2.3	2.9 ± 2.3
Hb * , mean ± SD		8.6 ±0.8	8.6 ±0.8	9.0 ±0.8	9.1 ±0.8	NA	NA
Active bleeding, No. (%)		246 (14.1)	213 (12.1)	36 (10.5)	49 (15.1)	NA	NA
pRBC, mean ± SD		0.7 ±1.6	2.5 ±2.3	2.9 ±3.7	2.8 ±2.7	1.6 ± 2.0	2.5 ±1.3

* Hb in the CRIT trial was estimated as Ht/3.39 All studies assumed p< 0.05 as statistical significance. AMI: acute myocardial infarction; CABG: coronary-artery bypass grafting; ESRD: end-stage renal disease; Hb: hemoglobin; NA: not available; LVEF: left ventricular ejection fraction; MI: myocardial infarction; NSTEMI: non-ST-segment elevation myocardial infarction; pRBC: packed red blood cell; PCI: percutaneous coronary intervention; Q1 and Q3, first and third quartiles; RCT: randomized controlled trial; RBC: red blood cell; SD: standard deviation; STEMI: ST-segment elevation myocardial infarction.

### Efficacy endpoints

In patients with AMI-associated anemia, there were no statistically significant differences between restrictive and liberal strategies for all-cause mortality ( Figure 2A ), cardiovascular mortality ( Figure 2B ), recurrent MI ( Figure 2C ), acute HF ( Figure 2D ), unscheduled revascularization ( Figure 2E ), stroke ( Figure 2F ), and acute kidney injury ( Figure 2G ).

**Figure 2:** Outcomes of restrictive versus liberal transfusion strategy in patients with acute myocardial infarction and anemia in 30 days. Caption: All-cause mortality (A), cardiovascular mortality (B), recurrent myocardial infarction (C), acute heart failure (D), unplanned revascularizations (E), stroke (F), and acute kidney injury (G); CRIT, Conservative Versus Liberal Red Cell Transfusion in Acute Myocardial Infarction (the CRIT Randomized Pilot Study);12 MINT: Restrictive or Liberal Transfusion Strategy in Myocardial Infarction and Anemia;13 REALITY: Effect of a Restrictive vs Liberal Blood Transfusion Strategy on Major Cardiovascular Events Among Patients With Acute Myocardial Infarction and Anemia.6 All studies assumed p< 0.05 as statistical significance.

The results were robust and consistent with the primary results when a leave-one-out analysis was performed for all-cause mortality, recurrent MI, and HF (Supplementary material eFigure 1 ).

In an additional assessment of the heterogeneity across studies, we evaluated the total transfusion used in each study. While the liberal strategy group showed a similar usage of transfusion in all trials, there was a high heterogeneity of treatment in the restrictive group. The mean RBC units in the MINT trial, CRIT, and REALITY were 0.7 (±1.6), 1.6 (±2.0), and 2.9 (±3.7), in the restrictive group respectively ( Table 1 , Figure 3A ).^6 , 12 , 13^In this last study, it is worth noting that the total number of packed RBC units administered in the restrictive group was even higher than that in the liberal strategy (342 vs 324) ( Figure 3A ).^6^This finding is significantly associated with the studies’ outcomes as there is a higher observed mortality in studies prescribing fewer packed RBC units in the restrictive group ( Figure 3B ). This finding likely explains the heterogeneity across studies.

**Figure 3:** Mean packed red blood cell transfused units (g/dL) difference (A) and mortality trend (B) in restrictive versus liberal transfusion strategy in patients with acute myocardial infarction and anemia in 30 days. CRIT, Conservative Versus Liberal Red Cell Transfusion in Acute Myocardial Infarction (the CRIT Randomized Pilot Study);12 MINT: Restrictive or Liberal Transfusion Strategy in Myocardial Infarction and Anemia;13 REALITY: Effect of a Restrictive vs Liberal Blood Transfusion Strategy on Major Cardiovascular Events Among Patients With Acute Myocardial Infarction and Anemia;6All studies assumed p< 0.05 as statistical significance.

### Safety endpoints

There were no differences between groups for severe allergic reaction, infection, acute lung injury or respiratory failure ( Figure 4 ).

**Figure 4:** Safety endpoints of restrictive versus liberal transfusion strategy in patients with acute myocardial infarction and anemia in 30 days. Caption: Allergic reactions (A), infections (B), and acute lung injury or respiratory failure (C). Trials abbreviations same as Figure 2. All studies assumed p< 0.05 as statistical significance.

### Quality assessment and risk of bias

The risk of bias for each of the five domains examined (selection, performance, detection, attrition, and reporting biases) was low and concordant between the two authors (L.M. and R.F.). The risk was assessed by RoB 2 tool for the primary and secondary endpoints considering intention-to-treat groups for each of the three included trials, resulting in an overall risk of bias for each study (Supplement material eTable 4 ).

The final level of evidence certainty for the pooled estimated effect (restrictive vs. liberal) was moderate for all-cause mortality, recurrent MI, and acute HF – outcomes reported in all included RCTs. For each of these outcomes, imprecision drove the downgrade given the potential benefit and potential harm within the Heart failure (Supplementary material eTable 5 ).

### Trial sequential analysis

In the TSA of all-cause mortality, the cumulative Z-curve did not surpass the monitoring boundaries, including futility, nor did the total sample size reach the required information size line ( Figure 5 ). Overall, these findings indicate absence of statistically significant difference in the pooled analysis and, thus far, they are insufficient to definitively exclude the possibility of an effect of a restrictive vs. liberal transfusion strategy for patients with AMI and anemia.

**Figure 5:** Trial Sequence Analysis of Restrictive vs liberal transfusion strategy on all-cause mortality within 30 days in patients with acute myocardial infarction and anemia. CRIT, Conservative Versus Liberal Red Cell Transfusion in Acute Myocardial Infarction (the CRIT Randomized Pilot Study); MINT: Restrictive or Liberal Transfusion Strategy in Myocardial Infarction and Anemia; REALITY, Effect of a Restrictive vs Liberal Blood Transfusion Strategy on Major Cardiovascular Events Among Patients With Acute Myocardial Infarction and Anemia.

## Discussion

In the present study, we found no significant differences in 30-day outcomes between a restrictive and liberal transfusion strategy for all-cause mortality, cardiovascular mortality, recurrent MI, unscheduled revascularization, HF, stroke, and acute kidney injury in patients with acute MI and anemia. Similarly, there were no significant differences in safety endpoints. These results remained robust in leave-one-out sensitivity analyses for all outcomes. However, there was a notable heterogeneity in the results across studies which seems to be at least partially explained by the differences in the amount of packed red blood cells used in the restrictive group across trials.

The restrictive approach mitigates the use of a limited and crucial resource and reduces potential risks for associated side effects. However, hypothesized benefit of maintaining higher Hb levels to increase oxygen availability to the ischemic myocardial area is plausible and justifies additional investigation though some studies suggest oxygen delivery might not be increased by transfusions.^25^The accumulated evidence suggests that a restrictive transfusion strategy is safe in a range of clinical scenarios,^1^and our current analysis supports this by documenting no differences in any of the safety endpoints between the two groups.

While no distinguishable differences in safety outcomes would suggest that either strategy would be routinely acceptable, the logistics of blood-derived products are more complex than most routinely used therapies. Blood resources are scarce, and any potential reduction in use can have a significant impact from a societal perspective as those resources can be directed to other patients in need.^26 – 28^This reallocation of scarce resources can also lead to cost savings as well as logistical improvements in the implementation of their use.^26^Thus, unless there is a proven benefit of more liberal transfusion strategies, a restrictive strategy would be more likely beneficial to society as a whole as no distinguishable clinical impact is noted for individual patients.

However, given the profound impact of those findings on routine clinical practice in cardiac intensive care units across the world, a more granular analysis is needed to allow for adequate implementation of current evidence. Within this context, our results provide novel insightful findings in the assessment of heterogeneity across the studies included in the present meta-analysis. While the treatment in the liberal strategy was impressively consistent across trials, there was substantial variability in the use of transfusion in what was defined as the "restrictive transfusion group". This difference was nontrivial as it varied from an average of less than 1 unit of packed RBC per patient to as high as almost three units. Some of this heterogeneity may be explained by the posttransfusion Hb targets in the restrictive group across trials. The MINT trial^13^did not require transfusion when Hb < 8 g/dL, as a result, it was the trial with the lowest mean units of blood transfused. On the contrary, the REALITY trial^6^had the highest upper limit posttransfusion target in the restrictive group (8 – 10 g/dL) which approximates to the posttransfusion target in the liberal group (≥ 10 g/dL) in the MINT and CRIT trials.^12 , 13^

Another point to be weighted is the duration of follow-up. In the REALITY trial,^6^despite the observed benefit of the restrictive strategy in the short term, the positive findings were not sustained at one year of follow up.^29^This finding reinforces the need for a well-defined sequential follow-up period, stratified by specific populations. Until then, the threshold to transfuse should be individualized, taking into consideration the patient's clinical context.

The difference in transfusion between the two groups in each trial is a key parameter to be explored as therapy can only be proven to show efficacy if its use is consistently and meaningfully different between the two study groups. If the control (restrictive) group receives almost as many transfusions as the treatment (liberal) group, no difference in outcomes should be expected. In our current analysis, we were able to show a direct correlation between the transfusion in the control arm and the observed outcomes (mortality rate reduction with an increase in mean units of blood transfused in the restrictive control group). While the analysis is limited by the small number of studies (three), the small sample size mainly leads to a considerably lower power in the analysis, but it would not have a substantial impact on the rate of false positive findings.

There are other potential explanations for the findings that we were unable to explore such as the heterogeneity among patients with AMI and anemia, along with the impracticality of considering Hb as an optimal surrogate for oxygen availability.^30 – 34^Also, AMI encompasses patients with STEMI and NSTEMI, who often exhibit distinct ischemic burdens, clinical severity, and 30-day prognosis, contributing to significant within-group heterogeneity.^30^Similarly, individuals with acute and chronic anemia may undergo different physiological adaptations to ischemia and blood transfusion, including variations in the optimal Hb-oxygen dissociation curve.^31 , 34^Within the same aspect, RBC changes, collectively referred to as ‘storage lesion’, may impact RBCs’ oxygen transport and tissue delivery differently in distinct patients, including the potentially distinct physiological implications of RBCs from different RBCs’ donors.^35 , 36^Additionally, it is plausible that patients with concurrent advanced chronic kidney disease may have a different response to ischemia and transfusion.^34 , 37^Finally, given the heterogeneous pathophysiological nature of AMI-associated anemia and possible complex and unbalanced effects on the intervention itself (blood transfusion), these two transfusion strategies may have different and opposing effects on distinct subgroups, which may not be fully appreciated due to the known power limitations of subgroup analyses.^38^While all those other alternative explanations for the findings are plausible and potentially important, none of them conflicts or contradicts our current findings.

When these findings about heterogeneity are contextualized with our TSA it becomes clear that current evidence is insufficient to clearly support one of the two strategies. However, our results provide guidance on how future studies in the field should be conducted to provide meaningful information for implementation of results. These studies should not only focus on adequate implementation of the treatment strategy in the liberal arm, but also perform a controlled implementation of transfusion in the restrictive arm to allow for a real difference in treatment between groups.

It is worth noting that the required information size from the TSA suggests that 77,885 patients would be needed to conclusively address this clinical question. This raises concerns that even if there was a significant difference between the two strategies, its effect would likely be small. However, this analysis is constrained by the cumulative sum analysis of the data of all included trials. If a difference between treatments was assumed, like that reported in the MINT trial (a three-fold increase in blood use), the calculated differences in outcomes would lead to a much smaller sample size requirement.

This study has limitations. First, all three RCTs were open-label due to the nature of the intervention (blood transfusion). Second, the absence of individual patient-level data precluded specific subgroup analyses. And third, the three studies used slightly different transfusion targets.

## Conclusion

In this meta-analysis of RCTs of patients with AMI and anemia, there was no statistical difference between restrictive and liberal transfusion strategies within 30 days for all-cause mortality, cardiovascular mortality, recurrent MI, unscheduled revascularization, acute heart failure, stroke, acute kidney injury, severe allergic reaction, infection, acute lung injury or respiratory failure. However, observed heterogeneity in transfusion use in the control (restrictive transfusion) groups likely explains at least part of the variability in outcomes across trials. The current results inform how to better implement future trials to explore this question.

## *Supplemental Materials

For additional information, please click here .
